# A CRISPR/Cas9-Based Mutagenesis Protocol for *Brachypodium distachyon* and Its Allopolyploid Relative, *Brachypodium hybridum*

**DOI:** 10.3389/fpls.2020.00614

**Published:** 2020-05-20

**Authors:** Karolina Hus, Alexander Betekhtin, Artur Pinski, Magdalena Rojek-Jelonek, Ewa Grzebelus, Candida Nibau, Mingjun Gao, Katja E. Jaeger, Glyn Jenkins, John H. Doonan, Robert Hasterok

**Affiliations:** ^1^Plant Cytogenetics and Molecular Biology Group, Institute of Biology, Biotechnology and Environmental Protection, Faculty of Natural Sciences, University of Silesia in Katowice, Katowice, Poland; ^2^Department of Plant Biology and Biotechnology, Faculty of Biotechnology and Horticulture, University of Agriculture in Cracow, Cracow, Poland; ^3^National Plant Phenomics Centre, Institute of Biological, Environmental and Rural Sciences, Aberystwyth University, Aberystwyth, United Kingdom; ^4^Sainsbury Laboratory, University of Cambridge, Cambridge, United Kingdom; ^5^Department for Plant Adaptation, Leibniz Institute of Vegetable and Ornamental Crops, Großbeeren, Germany; ^6^Institute of Biological, Environmental and Rural Sciences, Aberystwyth University, Aberystwyth, United Kingdom

**Keywords:** CRISPR/Cas9 system, targeted mutagenesis, *Brachypodium distachyon*, *Brachypodium hybridum*, *Agrobacterium*-mediated transformation, transient protoplast assay

## Abstract

The CRISPR/Cas9 system enables precise genome editing and is a useful tool for functional genomic studies. Here we report a detailed protocol for targeted genome editing in the model grass *Brachypodium distachyon* and its allotetraploid relative *B. hybridum*, describing gRNA design, a transient protoplast assay to test gRNA efficiency, *Agrobacterium*-mediated transformation and the selection and analysis of regenerated plants. In *B. distachyon*, we targeted the gene encoding phytoene desaturase (PDS), which is a crucial enzyme in the chlorophyll biosynthesis pathway. The albino phenotype of mutants obtained confirmed the effectiveness of the protocol for functional gene analysis. Additionally, we targeted two genes related to cell wall maintenance, encoding a fasciclin-like arabinogalactan protein (FLA) and a pectin methylesterase (PME), also in *B. distachyon*. Two genes encoding cyclin-dependent kinases (CDKG1 and CDKG2), which may be involved in DNA recombination were targeted in both *B. distachyon* and *B. hybridum*. Cas9 activity induces mainly insertions or deletions, resulting in frameshift mutations that, may lead to premature stop codons. Because of the close phylogenetic relationship between *Brachypodium* species and key temperate cereals and forage grasses, this protocol should be easily adapted to target genes underpinning agronomically important traits.

## Introduction

Precise and efficient genome targeting technologies are invaluable for functional genomics. Zinc-finger nucleases (ZFNs) (Miller et al., 2007; Caroll, 2008; Sander et al., 2011) and transcription activator–like effector nucleases (TALENs) (Christian et al., 2010; Miller et al., 2011) have been widely used to edit the genomes of many plant species (Shukla et al., 2009; Townsend et al., 2009; Zhang et al., 2010, 2013; Li et al., 2012; Wendt et al., 2013), including that of the model grass *Brachypodium distachyon* (Shan et al., 2013). However, the routine use of both ZFNs and TALENs is limited by a number of technically challenging steps including bespoke protein design and the relatively high cost of protein synthesis.

The CRISPR/Cas9 system (Jinek et al., 2012; Cong et al., 2013) revolutionized targeted mutagenesis and enables highly flexible genome editing at low cost. CRISPR/Cas9 technology was developed from the bacterial type II CRISPR/Cas system. The CRISPR-associated protein Cas9 is an endonuclease that uses a 20-nucleotide guide sequence (gRNA) within an RNA duplex, tracrRNA:crRNA to induce site-specific DSBs in DNA. To simplify genome editing, a dual tracrRNA:crRNA was engineered as a single guide RNA (sgRNA). Thus, only two components – Cas9 endonuclease and sgRNA – are required for targeted mutagenesis. Target site recognition by Cas9 is programmed by a sgRNA that encodes a gRNA sequence complementary to a target sequence in the DNA, but also requires recognition of a short neighboring sequence downstream of the 20-nucleotide target, known as the protospacer adjacent motif (PAM). The most commonly used variant of the Cas9 protein is an endonuclease from *Streptococcus pyogenes* (SpCas9) that requires the 20-nucleotide long sequence in the target DNA to be contiguous to a NGG PAM (5′-N_20_-NGG-3′). The 10–12 base pairs (bp) adjacent to the PAM at the 3′ end of the gRNA, called the “seed sequence,” determine Cas9 specificity and are generally more important than the rest of the gRNA sequence. Single mismatches between the target sequence and the gRNA can be tolerated but only in the “non-seed” region. DSBs are always introduced three nucleotides upstream of the PAM, so this method is very predictable and accurate. Notably, the CRISPR/Cas9 system does not rely upon expensive protein design and synthesis since the target sequence is recognized by a gRNA, which can be easily synthesized and incorporated into the sgRNA.

The development of efficient transformation protocols for *Brachypodium* (Draper et al., 2001; Christiansen et al., 2005; Pacurar et al., 2008; Vain et al., 2008; Vogel and Hill, 2008; Alves et al., 2009) has enabled the production of many publicly available T-DNA insertional lines (Bragg et al., 2012; Hsia et al., 2017) that can be useful in plant functional genomic studies. However, the T-DNA is inserted more or less randomly into the genome and may miss the genes of interest, particularly small ones. In contrast, the CRISPR/Cas9 system enables precise mutagenesis of virtually every selected gene. Many plant species have been successfully edited using this technique, such as *Arabidopsis thaliana*, tobacco, sorghum, rice (Jiang et al., 2013), wheat (Upadhyay et al., 2013), tomato (Pan et al., 2016) and watermelon (Tian et al., 2017). Although genome editing of *B. distachyon* has been reported before in several recent studies (O’Connor et al., 2017; Raissig et al., 2017; van der Schuren et al., 2018; Gao et al., 2019; Qin et al., 2019), none has presented a step-by-step protocol sufficiently detailed for use in the laboratory. Moreover, only *B. distachyon* in the genus has been edited to date.

Here we present a detailed step-by-step protocol for CRISPR/Cas9-mediated targeted mutagenesis of both a diploid (*B. distachyon*) and an allotetraploid species (*B. hybridum*). As a simple visual marker to gauge success of the editing, we chose as a target the gene encoding phytoene desaturase (PDS). *PDS* encodes one of the key enzymes involved in the chlorophyll biosynthesis pathway and, if knocked out, produces an albino phenotype (Shan et al., 2014; Pan et al., 2016; Tian et al., 2017; Kaur et al., 2018). To demonstrate that our protocol has general applicability, we also targeted two genes responsible for cell wall maintenance, encoding fasciclin-like arabinogalactan protein (FLA) and pectin methylesterase (PME) and 2 protein kinase genes from the CDKG group. We have previously found that expression of the *FLA* gene is upregulated in *B. distachyon* leaves in response to the temperature stress (Pinski et al., 2019). The inactivation of the second gene, *PME*, in *A. thaliana* led to increased susceptibility to salt stress (Yan et al., 2018). The Arabidopsis CDKG genes are also involved in modulating stress responses (Ma et al., 2015; Chen et al., 2018) and, moreover, are closely related to the CDK-like genes present at the Ph1 locus of *Triticum aestivum*, which is important for the restriction of pairing and recombination to homologous chromosomes only (Al-Kaff et al., 2008). In Arabidopsis, the *CDKG1* gene controls chromosome pairing during meiotic division in this diploid species (Zheng et al., 2014). The role of CDKG in homologous chromosome pairing in a polyploid species is not known, prompting us to target *CDKG* genes in both the diploid *B. distachyon* and its allotetraploid relative *B. hybridum*.

## Materials and Methods

### Plant Material and Growth Conditions

Seeds of *B. distachyon* reference genotype Bd21 and *B. hybridum* reference genotype ABR113 were cultivated in pots filled with 3:1 soil vermiculite mix in a greenhouse at 21 ± 1°C/16h-photoperiod. The 4-week-old plants were vernalised for two weeks at 4°C to induce flowering.

### Reagents

• 10xTris-borate-EDTA buffer (TBE; PanReac AppliChem, Cat. No. A0972,1000)
• 2,4-Dichlorophenoxyacetic acid (2,4-D; Sigma, Cat. No. 07299)
• 2-Mercaptoethanol (Sigma, Cat. No. M3148)
• Acetosyringone (Sigma, Cat. No. D134406)
• Agarose (VWR, Cat. No. 443666A)
• Agrobacterium Plasmid Miniprep DNA Purification Kit (EurX, Cat. No. E3535)
• *Agrobacterium tumefaciens* AGL1 strain
• Ampicillin (Duchefa, Cat. No. A01104)
• Biotin (Duchefa, Cat. No. B0603)
• BSA (Sigma, Cat. No. A7030)
• *Bsa*I restriction enzyme (New England BioLabs, Cat. No. R0535S)
• Calcium chloride (CaCl_2_; POCH, Cat. No. 874870116)
• Calcium hypochlorite (Ca(ClO)_2_; Sigma, Cat. No. 211389)
• Cefotaxime (Biotaksym, Polpharma)
• Cellulase (Duchefa, Cat. No. C8001)
• Cetyltrimethylammonium bromide (CTAB; Sigma, Cat. No. H6269)
• Chloroform (CHEMPUR, Cat. No. 112344305)
• Color Taq PCR Master Mix (2x) (EurX, Cat. No. E2525-01)
• Copper sulfate pentahydrate (CuSO_4_⋅5H_2_O; POCH, Cat. No. 658310116)
• Cysteine (Duchefa, Cat. No. C0706)
• D-Mannitol (Sigma, Cat. No. M947)
• DNA Electrophoresis Sample Loading Dye (Bio Rad, Cat. No. 1660401EDU)
• DNeasy Plant Mini Kit (Qiagen, Cat. No. 69106)
• Driselase (Sigma, Cat. No. D8037)
• Ethanol (POCH, Cat. No. 396480111)
• Ethylenediaminetetraacetic acid (EDTA; Sigma, Cat. No. T1503)
• Gateway LR Clonase II Enzyme mix (Thermo Fisher Scientific, Cat. No. 11791020)
• Gelrite (Duchefa, Cat. No. G1101)
• Glycine (Sigma, Cat. No. G7403)
• HEPES (Sigma, Cat. No. H3375)
• Hydrogen chloride (HCl; Merck, Cat. No. 1099110001)
• Hygromycin B (Duchefa, Cat. No. H0192)
• IPTG (Thermo Fisher Scientific, Cat. No. R0391)
• Isoamyl alcohol (POCH, Cat. No. 485560111)
• Isopropanol (CHEMPUR, Cat. No. 117515002)
• Kanamycin (Ducherfa, Cat. No. K0126)
• Kinetin (Sigma, Cat. No. K0753)
• L-glutamine (Duchefa, Cat. No. G0708)
• LB Agar (LabEmpire, Cat. No. LBL406.1)
• LB Broth (Sigma, Cat. No. L3022)
• Macerozyme R10 (Duchefa, Cat. No. M8002)
• Magnesium chloride (MgCl_2__;_ GLENTHAM, Cat. No. GK5046)
• Magnesium sulfate heptahydrate (MgSO_4_⋅7H_2_O; POCH, Cat. No. 613780111)
• Maxima H Minus First Strand cDNA Synthesis Kit (Thermo Fisher Scientific, Cat. No. K1651)
• MES (Sigma, Cat. No. M2933)
• Micro agar (Duchefa, Cat. No. M1002)
• Murashige and Skoog Basal Salt Mixture (MS; Duchefa, Cat. No. M0221)
• Myo-inositol (Duchefa, Cat. No. 10609)
• Nicotinic acid (Sigma, Cat. No. N-0765)
• NucleoSpin Gel and PCR Clean-up (Macherey-Nagel, Cat. No. 740609.10)
• O’GeneRuler 1 kb DNA Ladder (Thermo Fisher Scientific, Cat. No. SM1163)
• One Shot *ccd*B Survival 2 T1 Competent Cells (Thermo Fisher Scientific, Cat. No. A10460)
• One Shot TOP10 Chemically Competent *E. coli* (Thermo Fisher Scientific, Cat. No. C404003)
• PEG-4000 (Sigma, Cat. No. 81240)
• pGEM-T Vector System I (Promega, Cat. No. A3600)
• Phytagel (Sigma, Cat. No. P8169)
• Platinum^TM^ Taq DNA Polymerase High Fidelity (Thermo Fisher Scientific, Cat. No. 11304011)
• Potassium chloride (KCl; CHEMPUR, Cat. No. 117397402)
• Potassium dihydrogen phosphate (KH_2_PO_4_; POCH, Cat. No. 742020112)
• Potassium hydroxide (KOH; POCH, Cat. No. 746800113)
• Pyridoxine hydrochloride (Pyridoxine-HCl; Serva, Cat. No. 33990)
• QIAprep Spin Miniprep Kit (Qiagen, Cat. No. 27104)
• Ribonuclease A (RNase A; Sigma, Cat. No. R4875)
• Rifampicin (Bioshop, Cat. No. RIF2221)
• RNA isolation kit (Macherey-Nagel, Cat. No. 40120.50)
• RNase-Free DNase Set (Qiagen, Cat. No. 79254)
• RNeasy Mini Kit (Qiagen, Cat. No. 74104)
• SDS (Sigma, Cat. No. L3771)
• Sodium chloride (NaCl; POCH, Cat. No. A4121116)
• Sodium hydroxide (NaOH; Merck, Cat. No. 1099130001)
• Spectinomycin (Duchefa, Cat. No. S0188)
• Sucrose (CHEMPUR, Cat. No. 11720907)
• T4 DNA Ligase (New England BioLabs, Cat. No. M0202S)
• T7 Endonuclease I (New England BioLabs, Cat. No. M0302S)
• Thiamine hydrochloride (Thiamine-HCl; Sigma, Cat. No. T-3902)
• Timentin (Duchefa, Cat. No. T0190)
• Trizma hydrochloride (Tris–HCl; Sigma, Cat. No. T5941)
• Tryptone (Sigma, Cat. No. T-9410)
• Vectors pENTRsgRNA and pOsCas9 are from Miao et al. (2013)
• Vectors pYPQ131C, pYPQ132C, pYPQ142, pMDC32 and pYPQ167 are from Lowder et al. (2015)
• X-GAL (Thermo Fisher Scientific, Cat. No. R0404)
• Yeast extract (Sigma, Cat. No. 4-1625)

### Equipment

• 6-well plates
• Aluminum foil
• Autoclave
• Centrifuge for 5 mL tubes
• Centrifuge for 50 mL round bottom tubes
• Constant temperature (37°C) incubator
• Tissue culture room with controllable temperature and illumination (25 ± 1°C, 16 h/8 h light/dark photoperiod)
• Desiccator
• Disposable sterile plastic syringes
• Disposable sterile syringe filters (0.45 μm)
• DNA gel electrophoresis system
• Electroporator (capacitance 50 mF, load resistance 200 Ω, maximum power 25 W, current 25 mA and voltage 1800 V) with electroporation cuvettes (1 mm PKG 50, Cat. No. 1652089)
• Filter paper (9-cm diameter)
• Fine forceps
• Fridge (4°C) and freezers (−20°C and −80°C)
• Glass beakers (20 mL, 500 mL)
• Glass bottles (100 mL – 1 L)
• Glass Pasteur pipettes (230 mm)
• Glass Petri dishes (9-cm diameter)
• Glass test tubes (12 mL)
• Greenhouse for whole plant culture with controllable temperature and illumination (20 ± 1°C, 16 h/8 h light/dark photoperiod)
• Haemocytometer
• Laboratory microscope with 20x and 40x phase-contrast objectives
• Laminar flow hood with Bunsen burner
• Magnetic stirrer
• Microfuge for 0.2 mL – 2.0 mL tubes
• Microfuge tubes (0.2 mL, 0.5 mL, 1.5 mL and 2.0 mL)
• Micropipettes and corresponding pipette tips (10 μL – 5 mL)
• Microscope slides
• NanoDrop spectrophotometer (A260/280 and A260/230)
• Nylon filter (100 μm)
• Orbital incubator shaker (26 – 37°C; 30 – 250 rpm)
• Parafilm
• PCR thermal cycler
• pH meter
• Pipetting aid (1–50 mL)
• Plastic pestles
• Pots
• Propagator lid
• Scalpels (22 mm)
• Soil
• Spectrophotometer
• Spreaders (L-shaped)
• Stereomicroscope with white light supply unit
• Sterile plastic disposable tubes (20 mL, 50 mL)
• Sterile plastic Pasteur pipettes (10 mL)
• Sterile plastic Petri dishes (9-cm diameter)
• Sterile plastic square Petri dishes (9 × 9 cm)
• Sterile plastic round bottom centrifuge tubes (50 mL)
• Thermomixer
• Trays
• Vacuum pump
• Vermiculite
• Vortex mixer
• Water bath (42°C and 55°C)

### Reagent Setup

• 10% SDS [100 mL]: 10 g SDS + up to 100 mL H_2_O, store at room temperature (RT)
• 2.4-D 1 mg/mL [50 mL]: 50 mg 2.4-D dissolve in 2 mL of pure ethanol, add 48 mL H_2_O; use fresh
• 5% calcium hypochlorite [50 mL] 2.5 g Ca(ClO)_2_ + up to 50 mL H_2_O; mix well, use fresh
• Acetosyringone stock solution 30 mg/mL: 30 mg acetosyringone + 1 mL 70% ethanol, cover with aluminum foil to prevent light exposure, use fresh
• Ampicillin stock solution 100 mg/mL: 500 mg ampicillin + 5 mL H_2_O; filter-sterilize, aliquot 1 mL in 1.5 mL microfuge tubes, store at −20°C
• Annealing buffer [100 mL]: 0.292 g NaCl + 1 mL 1M Tris–HCl pH 8.0 + 400 μL 0.25 M EDTA pH 8.0 + up to 100 mL H_2_O; filter sterilize, store at 4°C
• Annealing mix [50 μL]: (A) dilute the synthesized oligonucleotides to 100 μM in annealing buffer (B) 2.5 μL 100 μM forward oligonucleotide + 2.5 μl 100 μM reverse oligonucleotide + 45 μL annealing buffer
• B5 Vitamins stock solution [500 mL]: 0.05 g nicotinic acid + 0.5 g thiamine-HCl + 0.05 g pyridoxine-HCl + 5 g myo-inositol + up to 500 mL H_2_O; adjust pH to 5.8 with KOH, filter-sterilize, store at 4°C
• BdAGM [250 mL]: (A) 1.075 g MS + 2.5 g sucrose + 2.5 g mannitol + up to 250 mL H_2_O, adjust the pH with KOH to 5.5; autoclave, let the medium to cool down, store at RT (B) add 375 μL 30 mg/mL acetosyringone just before the use
• BdCCM [250 mL]: 1.075 g MS + 7.5 g sucrose + up to 250 mL H_2_O, adjust the pH with KOH to 5.8, add 0.625 μL 1 mg/mL 2.4-D and 0.5 g phytagel; autoclave, let the medium to cool down, add 2.5 mL M5 Vitamins and 500 μL 30mg/mL acetosyringone, pour ∼25 mL of medium into sterile Petri dishes, store at 4°C
• BdCIM [250 mL]: 1.075 g MS + 7.5 g sucrose + 150 μL 1mg/mL CuSO_4_⋅5H_2_O + up to 250 mL H_2_O, adjust the pH with KOH to 5.8, add 0.625 μL 1 mg/mL 2.4-D and 0.5 g phytagel; autoclave, let the medium to cool down, add 2.5 mL M5 Vitamins, pour ∼25 mL of medium into sterile Petri dishes, store at RT
• BdGM [500 mL]: 0.86 g MS + 5 g sucrose + up to 500 mL H_2_O, adjust the pH with KOH to 5.8, add 3 g gelrite; autoclave, let the medium to cool down, add 5 mL B5 Vitamins + 175 μL 320 mg/mL timentin, pour ∼20 mL to sterile 50 mL plastic disposable tubes, store at RT
• BdHygR [500 mL]: 2.15 g MS + 500 mL H_2_O, adjust the pH with KOH to 5.8, add 1 g phytagel; autoclave, let the medium to cool down, add 50 μL 500 mg/mL hygromycin B, pour ∼100 mL of medium into sterile square Petri dishes, store at 4°C
• BdRM [500 mL]: 2.15 g MS + 15 g sucrose + up to 500 mL H_2_O, adjust the pH with KOH to 5.8, add 1 g phytagel; autoclave, let the medium to cool down, add 5 mL M5 Vitamins + 100 μL 1 mg/mL kinetin + 500 μL 200 mg/mL cefotaxime + 469 μL 320 mg/mL timentin + 20 μL 500 mg/mL hygromycin B, pour ∼25 mL of medium into sterile Petri dishes, store at 4°C
• BdSM30 [500 mL]: 2.15 g MS + 15 g sucrose + 300 μL 1mg/mL CuSO_4_⋅5H_2_O + up to 500 mL H_2_O, adjust the pH with KOH to 5.8, add 1.25 mL 1 mg/mL 2.4-D and 1 g phytagel; autoclave, let the medium to cool down, add 5 mL M5 Vitamins + 500 μL 200 mg/mL cefotaxime + 469 μL 320 mg/mL timentin + 30 μL 500 mg/mL hygromycin B, pour ∼25 mL of medium into sterile Petri dishes, store at 4°C
• BdSM40 [500 mL]: 2.15 g MS + 15 g sucrose + 300 μL 1 mg/mL CuSO_4_⋅5H_2_O + up to 500 mL H_2_O, adjust the pH with KOH to 5.8, add 1.25 mL 1 mg/mL 2.4-D and 1 g phytagel; autoclave, let the medium to cool down, add 5 mL M5 Vitamins + 500 μL 200 mg/mL cefotaxime + 469 μL 320 mg/mL timentin + 40 μL 500 mg/mL hygromycin B, pour ∼25 mL of medium into sterile Petri dishes, store at 4°C
• Biotin stock solution 1 mg/mL: 50 mg biotin dissolve in 1 mL 1M KOH, add 49 mL H_2_O; filter sterilize, cover with aluminum foil to prevent light exposure, store at 4°C
• *Bsa*I restriction enzyme digestion of pENTRsgRNA vector [50 μL]: 1 μg pENTRsgRNA + 5 μL of *Bsa*I buffer + 1μL of *Bsa*I restriction enzyme + up to 50 μl H_2_O
• Cas9 PCR reaction [20 μL]: 10 μL Master Mix + 0.8 μL 10 μM forward primer + 0.8 μL 10 μM reverse primer + 1 μL isolated DNA + 7.4 μL H_2_O
• Cefotaxime stock solution 200 mg/mL: 1 g cefotaxime + 5 mL H_2_O; filter-sterilize, aliquot 1 mL in 1.5 mL microfuge tubes, store at −20°C
• CTAB buffer: (A) [100 mL] 2 g CTAB + 10 mL 1 M Tris–HCl pH 8.0 + 4 mL 0.5 M EDTA + 28 mL 5 M NaCl + up to 100 mL H_2_O; autoclave and store at RT (B) 5 mL (A) solution + 10 μL 2-Mercaptoethanol; preheat to 60°C, use fresh
• CuSO_4_⋅5H_2_O 1 mg/mL [50 mL]: 50 mg CuSO_4_⋅5H_2_O + up to 50 mL H_2_O, store at RT
• EDTA 0.25 M [100 mL]: 7.3 g EDTA + up to 100 mL H_2_O; add crystals of NaOH till pH 8.0, autoclave, store at 4°C
• EDTA 0.5 M [100 mL]: 14.6 g EDTA + up to 100 mL H_2_O; add crystals of NaOH till pH 8.0, autoclave, store at 4°C
• Enzyme solution (for protoplast isolation): (A) [100 mL]: 11.388 g mannitol + 0.15 g KCl + 0.11 g CaCl_2_ + 0.39 g MES + 0.01 g BSA + up to 100 mL H_2_O; adjust pH to 5.7 with KOH

**NOTE:** Dissolve mannitol thoroughly before adding other ingredients.

• (B) 20 mL (A) solution + 0.08 g macerozyme + 0.04 g cellulase + 0.1 g driselase; mix for 30 min with a magnetic stirrer and transfer to the 50 mL plastic disposable tube. Incubate 10 min in water bath at 55°C and then keep on ice for 10 min. Filter sterilize when adding to cut leaves, use fresh
• Extraction buffer [50 mL]: 10 mL 1 M Tris–HCl pH 8.0 + 12.5 mL 1 M NaCl + 5 mL 0.25 M EDTA pH 8.0 + 2.5 mL 10% SDS + up to 50 mL H_2_O, store at RT
• Kanamycin stock solution 50 mg/mL: 250 mg kanamycin + 5 mL H_2_O; filter-sterilize, aliquot 1 mL in 1.5 mL microfuge tubes, store at −20°C
• Kinetin stock solution 1 mg/mL [50 mL]: 50 mg kinetin dissolve in 1 mL 1N NaOH, add 49 mL H_2_O; filter sterilize, store at 4°C
• KOH 1 M [100 mL]: 5.6 g KOH + up to 100 mL H_2_O; store at RT
• KOH solution to adjust pH of culture media and to dissolve components 0.5 M [100 mL]: 2.8 g KOH + up to 100 mL H_2_O; store at RT
• LB agar plates + 100 μg/mL ampicillin [250 mL]: 8.75 g LB agar + 250 mL H_2_O; autoclave, let the medium to cool down, add 250 μL 100 mg/mL ampicillin, pour ∼25 mL of medium into sterile Petri dishes, store at 4°C
• LB agar plates + 50 μg/mL kanamycin [250 mL]: 8.75 g LB agar + 250 mL H_2_O; autoclave, let the medium to cool down, add 250 μL 50 mg/mL kanamycin, pour ∼25 mL of medium into sterile Petri dishes, store at 4°C
• LB agar plates + 50 μg/mL kanamycin + 25 μg/mL rifampicin [250 mL]: 8.75 g LB agar + 250 mL H_2_O; autoclave, let the medium to cool down, add 250 μL 50 mg/mL kanamycin and 62.5 μL 100 mg/mL rifampicin, pour ∼25 mL of medium into sterile Petri dishes, store at 4°C
• LB agar plates + 50 μg/mL spectinomycin [250 mL]: 8.75 g LB agar + 250 mL H_2_O; autoclave, let the medium to cool down, add 250 μL 50 mg/mL spectinomycin, pour ∼25 mL of medium into sterile Petri dishes, store at 4°C
• LB agar plates + 50 μg/mL spectinomycin + 25 μg/mL rifampicin [250 mL]: 8.75 g LB agar + 250 mL H_2_O; autoclave, let the medium to cool down, add 250 μL 50 mg/mL spectinomycin and 62.5 μL 100 mg/mL rifampicin, pour ∼25 mL of medium into sterile Petri dishes, store at 4°C
• LB liquid medium [250 mL]: 5 g LB Broth + 250 mL H_2_O; autoclave, store at 4°C
• LB liquid medium + 100 μg/mL ampicillin [250 mL]: add 250 μL 100 mg/mL ampicillin to 250 mL of RT LB liquid medium; store at 4°C
• LB liquid medium + 50 μg/mL kanamycin [250 mL]: add 250 μL 50 mg/mL kanamycin to 250 mL of RT LB liquid medium; store at 4°C
• LB liquid medium + 50 μg/mL kanamycin + 25 μg/mL rifampicin [250 mL]: add 250 μL 50 mg/mL kanamycin and 62.5 μL 100 mg/mL rifampicin to 250 mL of RT LB liquid medium; store at 4°C
• LB liquid medium + 50 μg/mL spectinomycin [250 mL]: add 250 μL 50 mg/mL spectinomycin to 250 mL of RT LB liquid medium; store at 4°C
• LB liquid medium + 50 μg/mL spectinomycin + 25 μg/mL rifampicin [250 mL]: add 250 μL 50 mg/mL spectinomycin and 62.5 μL 100 mg/mL rifampicin to 250 mL of RT LB liquid medium; store at 4°C
• Ligation of cut pENTRsgRNA vector [10 μL]: 1 μL cut pENTRsgRNA + 1 μL ligase buffer + 1 μL annealed oligonucleotides + 0.5 μL ligase + 6.5 μL H_2_O
• LR recombination mix [4 μL]: 1 μL pENTRsgRNA with the introduced gRNA sequence (10 ng/μL) + 1 μL pOsCas9 (109 ng/μL) + 2 μL TE buffer
• M5 Vitamins stock solution [500 mL]: 0.02 g nicotinic acid + 0.025 g thiamine-HCl + 2 g cysteine + 0.1 g glycine + 0.02 g pyridoxine-HCl + up to 500 mL H_2_O; adjust pH to 5.8 with KOH, filter-sterilize, store at 4°C

**NOTE:** Do not use the stock solution if you notice any precipitation.

• MGL + S50 + AS30 [500 mL]: 2.5 g tryptone + 1.25 g yeast extract + 2.6 g NaCl + 5 g mannitol + 1.16 g L-glutamine + 0.25 g KH_2_PO_4_ + 0.1 g MgSO_4_⋅7H_2_O + up to 500 mL H_2_O, adjust the pH with KOH to 7.2, add 5 g micro agar; autoclave, let the medium to cool down, add 1 μL 1 mg/mL biotin + 500 μL 50 mg/mL spectinomycin + 500 μL 30 mg/mL acetosyringone, pour ∼25 mL of medium into sterile Petri dishes, store at 4°C
• MMG solution [100 mL]: 7.29 g mannitol + 0.08 g MES + 0.14 g MgCl_2_ + up to 100 mL H_2_O; adjust pH to 5.7 with KOH, autoclave, store at RT
• NaCl 1 M [100 mL]: 5.84 g NaCl + up to 100 mL H_2_O; autoclave, store at 4°C
• NaCl 5 M [100 mL]: 29.22 g NaCl + up to 100 mL H_2_O; autoclave, store at 4°C
• NaOH 1 N [100 mL]: 4 g NaOH + up to 100 mL H_2_O; store at RT
• PEG-Calcium solution [100 mL]: 40 g PEG-4000 + 3.64 g mannitol + 1.1g CaCl_2_ + up to 100 mL H_2_O; filter sterilize, store at RT
• PSII solution [250 mL]: 22.771 g mannitol + up to 250 mL H_2_O; adjust pH to 5.6–5.8, autoclave, store at RT
• Rifampicin stock solution 100 mg/mL: 500 mg rifampicin + 5 mL 100% methanol; filter-sterilize, aliquot 1 mL in 1.5 mL microfuge tubes, store at −20°C
• RNase A solution 100mg/mL: (A) RNase buffer [50 mL]: 0.5 mL 1 M Tris–HCl pH 8.0 + 150 μL 5 M NaCl + up to 50 mL H_2_O; filter sterilize (B) 500 mg RNase A + 5 mL RNase buffer; heat to 100°C for 15 min, cool down at RT, store at −20°C
• Spectinomycin stock solution 50 mg/mL: 250 mg spectinomycin + 5 mL H_2_O; filter-sterilize, aliquot 1 mL in 1.5 mL microfuge tubes, store at −20°C
• TE buffer [100 mL]: 10 mL 1 M Tris–HCl pH 8.0 + 400 μL EDTA 0.25M + up to 100 mL H_2_O, store at 4°C
• Timentin stock solution 320 mg/mL: 1.6 g timentin + 5 mL H_2_O; filter-sterilize, aliquot 1 mL in 1.5 mL microfuge tubes, store at −20°C
• Tris–HCl 1 M [100 mL]: 12.1 g Tris + up to 100 mL H_2_O; add pure HCl till pH 8.0, autoclave, store at 4°C
• W5 solution [250 mL]: 2.25 g NaCl + 4.6 g CaCl_2_ + 0.0925 g KCl + 0.1 g MES + up to 250 mL H_2_O; adjust pH to 5.7 with KOH, autoclave, store at RT

Autoclaving conditions: (40 min total cycle, including 20 min at 120°C).

### Step-by-Step Procedure

**FIGURE 1:**
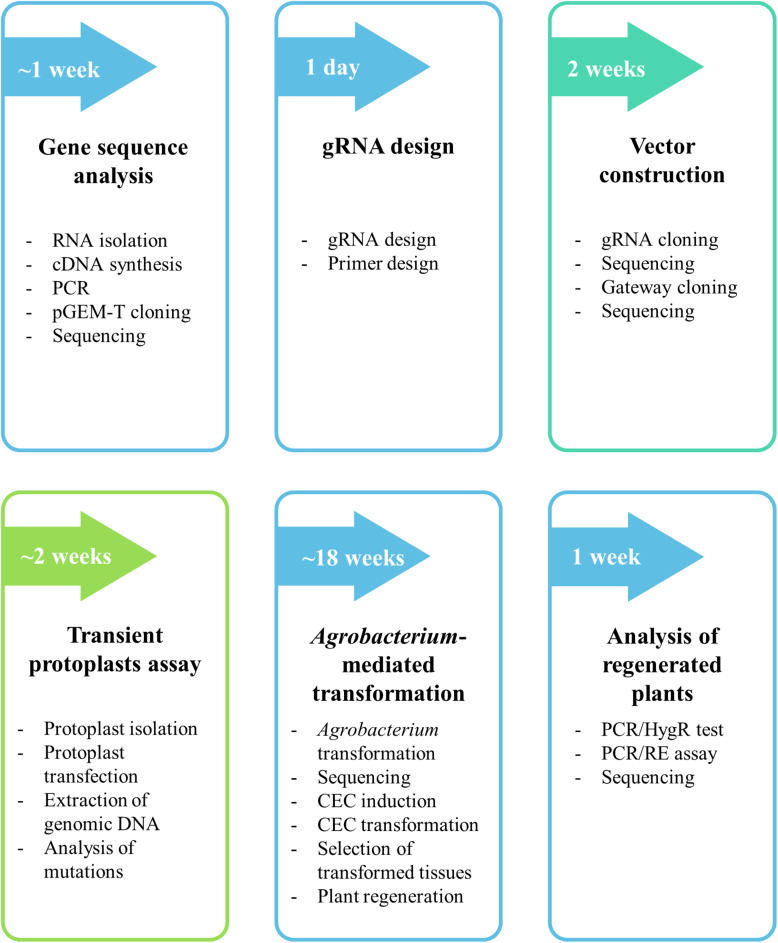
Flow chart of the protocol for targeted mutagenesis of *Brachypodium* species. The detailed procedure is found in the section “MATERIALS AND METHODS.”

The protocol described here consists of six major steps (gene sequence analysis, gRNA design, vector construction, transient protoplast assay, *Agrobacterium*-mediated transformation and analysis of regenerated plants) and is summarized in Figure 1. We describe each step in detail in the following sections.

#### Gene Sequence Analysis (Timing ∼1 Week)

Mismatches within the gRNA sequence can affect the endonucleolytic activity of the Cas9 protein, especially if they occur in the “seed region.” For this reason, it is advisable to sequence the target gene in the species or even the genotype to be edited.

(1). Isolate total RNA from leaves of the genotype to be edited using an RNA isolation kit (Macherey-Nagel) and following the manufacturer’s protocol. Treat with DNase (Qiagen) and purify the isolated RNA using an RNeasy Mini Kit (Qiagen).
(2). Check the concentration and quality of the isolated RNA using a NanoDrop spectrophotometer. The A260/280 and A260/230 values must be greater than 1.8. The concentration of the purified RNA should not be less than 100 ng/μL. Take 1000 ng of isolated RNA and add RNase-free water to obtain a volume of 10 μL. Add 2 μL of DNA Electrophoresis Sample Loading Dye (Bio Rad) and run on a 1% agarose gel to check for the quality and integrity. Intact total RNA should have two (28S and 18S rRNA) sharp bands. Use 1000 ng of RNA to synthesize cDNA using a Maxima H Minus First Strand cDNA Synthesis Kit according to the manufacturer’s instructions with oligo (dT)_18_ primer.
(3). Amplify the whole coding DNA sequence (CDS) of the gene using specific 18-mers corresponding to the beginning and the end of the sequence. Design the primers based on the sequence of CDS available for particular genes in the Phytozome database. Perform PCR using Platinum^TM^ Taq DNA Polymerase High Fidelity (Thermo Fisher Scientific) and following the manufacturer’s protocol, and run the products on a 1% agarose gel.

**NOTE:** The use of cDNA as a template allows analysis of the gene exon/intron structure. However, as an alternative for small genes, the amplification can be also performed using genomic DNA (gDNA) instead of cDNA. This can be particularly useful, if the expression of a target gene is not high enough. Genomic DNA can be isolated using DNeasy Plant Mini Kit (Qiagen).

(4). Extract the desired band from the gel using a NucleoSpin Gel and PCR Clean-up kit and ligate into a pGEM-T vector according to the manufacturer’s instructions.
(5). Transform the ligation (2 μL) into One Shot TOP10 Chemically Competent *Escherichia coli*, plate in plates LB with 100 μg/mL ampicillin and grow overnight at 37°C. Select single colonies and inoculate into 5 mL of LB with 100 μg/mL ampicillin, and incubate overnight at 37°C with constant shaking at 250 rpm.
(6). Isolate plasmids using a QIAprep Spin Miniprep Kit and sequence with an M13 primer pair (sequences of all primers used can be found in Supplementary Table S1). Compare the sequences obtained to those in an online database (for example in NCBI or Phytozome).

**NOTE:** Sequence the gene from five colonies.

**NOTE:** *B. hybridum* is an allotetraploid, resulting from natural hybridisation between *B. distachyon* and *B. stacei*. The alleles of a gene derived from the *B. distachyon* subgenome (Bd) may differ from the alleles derived from the *B. stacei* subgenome (Bs). Thus, if the goal is simultaneous mutagenesis of all copies of the gene in *B. hybridum*, it is important to obtain and compare the gene sequences from both ancestor genomes – Bd and Bs (at least five colonies for each).

#### gRNA Design (Timing – 1 Day)

Designing gRNA for diploid *B. distachyon* is much simpler than for allotetraploid *B. hybridum*, as the latter requires a target sequence which is conserved in both its Bd and Bs subgenomes. If it is not possible to find such a sequence, consider sequences with one mismatch at least 13 nucleotides away from PAM, i.e., outside of the gRNA “seed” sequence.

In general, a few important factors should be considered when designing gRNA:

• If the goal of the experiment is to knock out the gene, the target sequence should be within an exon, preferably the first.

**NOTE:** It is often not possible to identify a target sequence within the first exon. However, targeting another exon may also prove effective (see the example of *pds* mutants, section “RESULTS AND DISCUSSION”).

• The 20-nucleotide target sequence should immediately precede the 5′-NGG PAM. Target either sense (5′-N_(__20__)_-NGG-3′) or antisense (5′-CCN-N_(__20__)_-3′) strand of the DNA.
• Off-target sites that could potentially be edited using individual gRNAs should be identified, using the Cas-OFFinder online bioinformatic tool^1^ (Bae et al., 2014). Align all the potential gRNA sequences with the *B. distachyon* genome using the following criteria: (1) Mismatch number equal or less than 2 and bulge size equal or less than 1, (2) Mismatch number equal or less than 4 and bulge size 0. Avoid gRNA with potential off-target sites within an exon. Permissible off-target sites include introns, intergenic regions or mismatched sequences in the gRNA “seed” region.
• The target 20-nucleotide sequence should have a restriction site at the Cas9 cutting site (3-bp upstream 5′-NGG). This enables much faster and cheaper analysis of regenerated plants after transformation and allows the analysis of gRNA functionality in transient protoplast assays.

**NOTE:** Designing a gRNA with a restriction site is highly recommended. However, if the design of such a gRNA is impossible for a target gene, gRNA without a restriction site can be used. The protoplast test may be omitted and the analysis, whether the sequence is edited or not, can be performed using cloning and sequencing without a PCR/restriction enzyme digestion (PCR/RE) assay.

Optimal gRNA sequences can be selected manually or using bioinformatic tools, as for example https://www.genome.arizona.edu/crispr/CRISPRsearch.html or http://www.e-crisp.org/E-CRISP/. We designed all the gRNAs using the Geneious Prime program, that finds both potential 5′-N_(__20__)_-NGG-3′ sites and restriction sites.

(7). Design the optimal gRNA sequence with a restriction site at the Cas9 cutting site.
(8). Design primers for amplification of a part of the gene with the gRNA sequence. *B. hybridum* requires primers that are conserved in both Bd and Bs genomes.

**NOTE:** The restriction site in the gRNA target should be unique within the amplicon, if the intention is to use a PCR/RE assay to detect the mutations. The digestion of the PCR product using a relevant restriction enzyme should produce two smaller fragments of different size in order to be identified by gel electrophoresis.

#### Vector Construction (Timing ∼2 Weeks)

**FIGURE 2:**
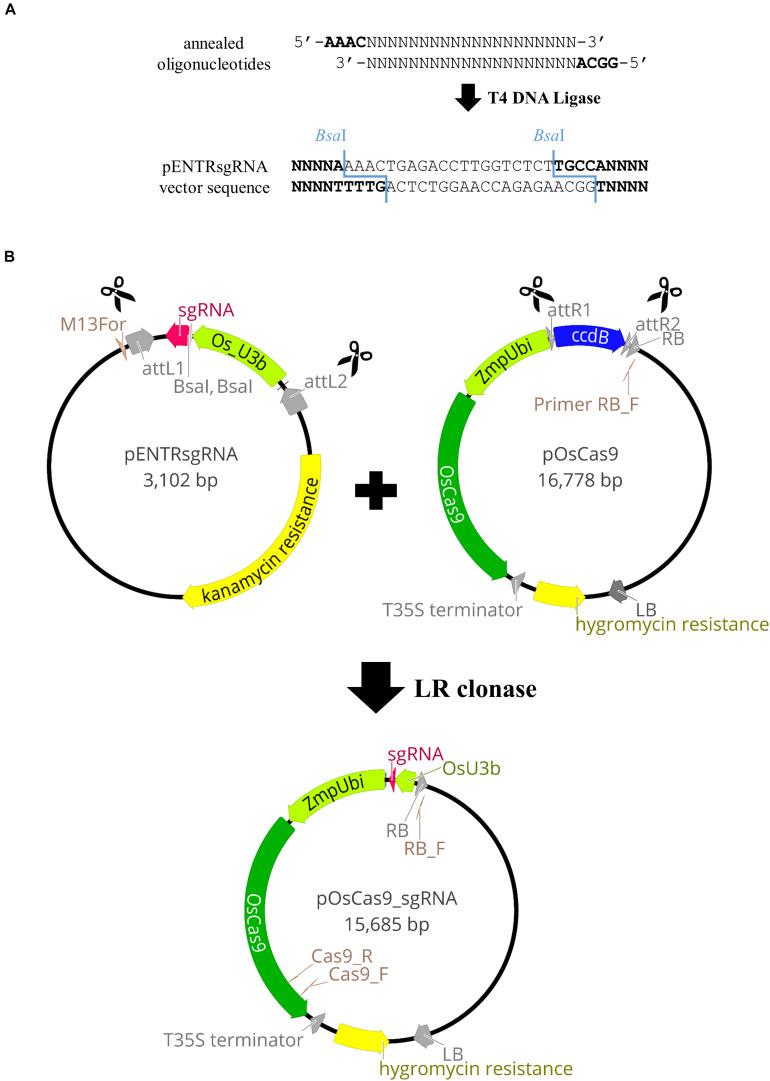
Schematic illustration of the pOsCas9_sgRNA vector construction. **(A)** Cloning of the gRNA sequence into pENTRsgRNA vector. **(B)** Diagrams of the plasmids used for targeted mutagenesis of *Brachypodium* species and the scheme of the LR recombination reaction.

**NOTE:** The pOsCas9 vector contains the *ccd*B gene and cannot be transformed into the One Shot TOP10 Chemically Competent *E. coli*. Consequently, it should be transformed into One Shot *ccd*B Survival 2 T1 Competent Cells.

Vectors with codon-optimized Cas9 and relevant gene promoters were used, as in targeted mutagenesis in rice (Miao et al., 2013). These comprise pENTRsgRNA containing the gRNA cloning site, and pOsCas9 with the gene encoding the Cas9 protein (detailed information about the vector structure can be seen in Figure 2B). The pENTRsgRNA vector carries a kanamycin resistance gene, and the pOsCas9 vector carries a spectinomycin resistance gene for antibiotic selection during plasmid cloning. The recommended working concentration for kanamycin and spectinomycin is 50 μg/mL.

For the targeted mutagenesis of the *PME* gene, a different set of vectors were prepared according to Lowder et al. (2015). These vectors can carry two different gRNAs and enable simultaneous editing in a single transformation experiment. pYPQ131C and pYPQ132C vectors carrying the U6 promoter from *Oryza sativa*, were used for the introduction of the gRNA. As the detailed protocol for the preparation of these vectors has been reported previously by Lowder et al. (2015), we will focus on the preparation of the mature vector from vectors pENTRsgRNA and pOsCas9.

(9). The pENTRsgRNA vector is linearised by *Bsa*I restriction enzyme digestion (Figure 2A). Prepare two *Bsa*I restriction enzyme digestion reactions (see section “Reagent Setup”) and incubate overnight in a PCR thermal cycler at 37°C. Inactivate the enzyme at 65°C for 20 min and stop the reaction by adding 10 μL of 6x gel loading dye to both 50 μL reactions. Run the reactions on a 0.7% agarose gel, extract the linearised plasmid, elute in 30 μL of EB and split into 3 aliquots to avoid freeze-thaw cycling.
(10). Once a target site is selected (step 7), forward and reverse oligonucleotides are designed and synthesized, consisting of a 20-nucleotide gRNA sequence (gRNA_F and gRNA_R) with 4-nucleotide adapters (bold) complementary to the overhangs obtained after *Bsa*I digestion of pENTRsgRNA (Figure 2A).
  5′-**GGCA**-gRNA_F-3′
  5′-**AAAC**-gRNA_R-3′
  Examples of oligonucleotides used in this study can be found in Supplementary Table S2.
(11). Anneal the oligonucleotides. Preheat block to 95°C and prepare the annealing mix (see section “Reagent Setup”). Incubate the reaction for 5 min at 95°C, transfer the tube to RT and let it cool down (∼45 min).

**NOTE:** Carefully remove the tube from 95°C to avoid opening the tube.

**NOTE:** Oligonucleotide annealing must be prepared immediately before the ligation.

(12). Prepare the ligation of the linearised pENTRsgRNA vector (from step 9) with an annealed oligonucleotides (from step 11) (see section “Reagent Setup”) and run overnight in a PCR thermal cycler using the following program: (10°C for 30 s; 30°C for 30 s) – 300 cycles and 12°C – ∞.
(13). Transform 1 μL of the ligation reaction into 25 μL of One Shot TOP10 Chemically Competent *E. coli* according to the manufacturer’s protocol. At the end of the transformation procedure spread 200 μL of transformation mix onto LB agar plates with kanamycin 50 μg/mL and incubate upside down overnight at 37°C. Then inoculate 5 single colonies into 5 mL of LB + kanamycin 50 μg/mL, grow overnight at 37°C with shaking at 250 rpm, isolate plasmids and sequence with the M13For primer to check for the presence and integrity of the sgRNA.
  Gateway^®^ Recombination Cloning Technology was used to combine the sgRNA sequence, together with the relevant promoter, to the destination vector containing the gene encoding the Cas9 protein. The LR recombination reaction was carried out between an *att*L-containing pENTRsgRNA with the incorporated gRNA sequence and an *att*R-containing pOsCas9 vector, producing the pOsCas9_sgRNA final vector (Figure 2B).
(14). The LR recombination reaction is effected by first diluting the pENTRsgRNA vector to 10 ng/μL, and the pOsCas9 plasmid to 109 ng/μL.

Prepare the LR recombination mix (see section “Reagent Setup”). Thaw on ice the LR Clonase II Enzyme Mix for about 2 min, vortex briefly and add 1 μL to the reaction mixture. Vortex briefly again, centrifuge and incubate at 25°C for 3 h. After incubation, add 0.5 μL of proteinase K solution, vortex briefly and incubate for 10 min at 37°C. Transform 0.5 μL of the LR reaction into 25 μL of One Shot TOP10 Chemically Competent *E. coli*, at the end of the transformation procedure spread 200 μL of transformation mix onto LB agar plates with spectinomycin at 50 μg/mL, and incubate upside down overnight at 37°C. Inoculate 5 single colonies into 5 mL of LB + spectinomycin at 50 μg/mL, grow overnight at 37°C with 250 rpm shaking, and isolate plasmids (elute in water instead of EB, as these plasmids will be used later for *Agrobacterium* transformation in step 23). Sequence isolated plasmids with the RB_F primer to check for the presence of the sgRNA. Plasmids containing the expected pOsCas9_sgRNA sequence are stored at −80°C as glycerol stocks after mixing 500 μL of the overnight liquid bacterial culture with 500 μL of 50% glycerol.

#### Transient Protoplast Assay (Timing ∼2 Weeks)

Before *Agrobacterium*-mediated transformation, vectors may be tested using a protoplast transient assay. An optimized, short protocol for transient protoplast assay in *Brachypodium* species is presented below. This test might be useful for checking the functionality of individual vectors in *Brachypodium* genome editing (see section “RESULTS AND DISCUSSION”). However, since false negative results can be also obtained, it should be emphasized, that the use of this test (step 15 – 22) is optional and it is also possible to skip it and go directly to *Agrobacterium*-mediated transformation (step 23).

(15). A high concentration of plasmid DNA is required for protoplast transfection (at least 1 μg/μL). Inoculate the glycerol stock of *E. coli* with the pOsCas9_sgRNA vector (prepared in step 14) in 5 mL LB + spectinomycin at 50 μg/mL. Grow overnight at 37°C with 250 rpm shaking and isolate plasmids as follows:

Centrifuge 5 mL of bacterial culture for 5 min at 8000 rpm and discard the supernatant. Use reagents from QIAprep Spin Miniprep Kit. Add 250 μL of P1 buffer and mix to resuspend the bacteria. Add 250 μL of P2 buffer and wait for 4.5 min, before mixing gently by inverting.

**NOTE:** To avoid contamination with bacterial genomic DNA, do not extend the incubation in P2 buffer.

Add 350 μL N3 buffer, mix by inverting and leave for 5 min on ice. Centrifuge for 10 min at 14 000 rpm. Transfer the clear supernatant to a clean 1.5 mL tube, taking care not to disturb the pellet, add 1 mL of cold (−20°C) isopropanol and mix thoroughly by inverting and centrifuge for 20 min at 14 000 rpm. Discard the supernatant and add 1 mL 70% ethanol, mix by inverting and centrifuge for 3 min at 14 000 rpm. Discard the supernatant and repeat the washing with ethanol. Discard the supernatant, air dry and resuspend in 30 μL of EB buffer. Check the concentration on a NanoDrop spectrophotometer and dilute the plasmid to a concentration of 1 μg/μL. Store at −20°C.

**FIGURE 3:**
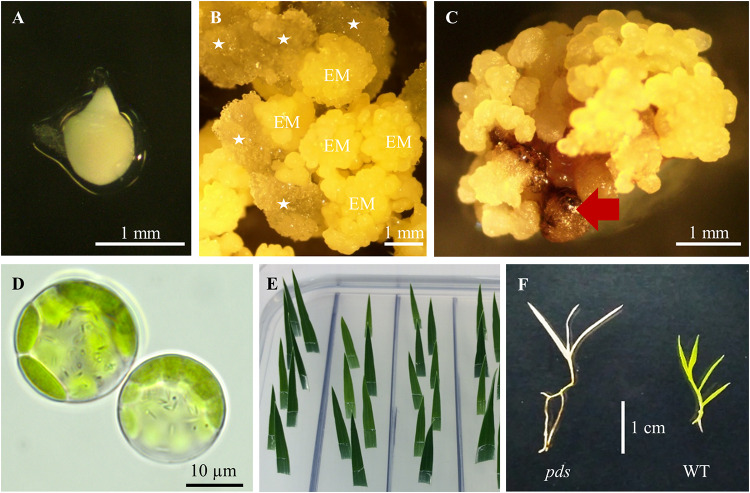
Tissue culture in *B. distachyon*. **(A)** Immature embryo used for callus induction. **(B)** Induced callus: EM – embryogenic masses; parenchymatous cells are marked by white asterisks. **(C)** Embryogenic callus after transformation. Non-transformed calli are indicated by the red arrow. **(D)** Two day-old protoplasts of *B. distachyon*. **(E)** HygR test with *B. distachyon* leaves. **(F)** Albino phenotype of the *B. distachyon*. *pds* mutant in comparison to the green wild-type (WT) plant.

A step-by-step protocol for protoplast isolation from leaf mesophyll cells of the Bd21-3 inbred line has been published by Jung et al. (2015). We present the optimized protocol for the isolation of protoplasts from *B. distachyon* Bd21. This protocol can be completed within one day and uses young leaves from 5–7 week-old Bd21 plants. To avoid contamination of the protoplasts, work under sterile conditions in a laminar flow hood, and use sterilized buffers and sterile Petri dishes, tubes, beakers, filters and Pasteur pipettes. Handle protoplasts with care, as they are easily broken.

(16). Place 0.2 g of young *B. distachyon* leaves in a glass Petri dish and add a small amount of PSII solution. Cut the leaves using two 22 mm scalpels into small (∼2 mm) pieces. Wash the plant material with ∼10 mL of PSII solution. Remove the PSII solution using a Pasteur pipette and add immediately the enzyme solution to avoid drying of the material. Mix gently and vacuum-infiltrate for 30 min at RT by placing the open Petri dish with the plant material in a desiccator under a laminar flow hood, running the vacuum pump up to 500 mBa, turning off the pump and holding the vacuum for 30 min. After 30 min close the Petri dish, seal with Parafilm and wrap in aluminum foil. Transfer the Petri dish to an orbital incubator shaker for 3 h with gentle shaking at 30 rpm at 26°C. After 3 h, increase the shaking speed to 50 rpm and continue the incubation for 30 min. Collect the protoplasts in round bottom centrifuge tubes by filtering through a nylon filter. To increase the protoplast yield, wash the Petri dish with 10 mL of W5 solution and filter it into the same round bottom tube. Centrifuge the protoplasts for 5 min at 1000 rpm and 4°C, remove the supernatant with a pipette (the protoplasts will be collected at the bottom of the tube), and slowly add 30 mL of W5 solution. Centrifuge for 5 min at 1000 rpm and 4°C. Pipette out the W5 solution and resuspend the purified protoplasts in 2 mL of W5 solution. Determine protoplast yield and integrity by cell counting using a haemocytometer. For optimal transformation, the density of protoplasts should be ∼10^6^ protoplasts/mL. If the density of the isolated protoplasts is higher, add more W5 solution.
(17). Incubate freshly isolated protoplasts for 30 min on ice, and centrifuge for 5 min at 1000 rpm and 4°C. Pipette out the supernatant and add an equal amount of MMG solution to obtain the required density of protoplasts. Make two transfection reactions for each vector. Prepare two 12 mL sterile glass test tubes and add 10 μL of 1 μg/μL plasmid to the bottom of each. Slowly add 100 μL of isolated protoplasts to each tube and mix gently. Initiate the transfection reaction by addition of 110 μL PEG solution and mix gently. Incubate for 7 min at RT. Terminate the reaction by adding 700 μL W5 solution, mix gently and centrifuge for 2 min at 1000 rpm and 4°C. Pipette out the supernatant to leave ∼50 μL of protoplasts suspension. Meanwhile, prepare plates for protoplast incubation. Use 6-well plates in which reactions can be incubated from three different plasmids (2 wells each). Transfer the protoplasts from each reaction to 1 mL of W5 solution in each well. Cover the plates with Parafilm and incubate for 2 days at 25°C in the dark.
(18). After 2 days of incubation, check the quality of the protoplasts using a light microscope. Most of them should still have a spherical shape (Figure 3D). If most protoplasts have broken, it is better to repeat the isolation and transfection experiments (steps 16 and 17). From step 18 onward there is no need to work under sterile conditions. Use the CTAB method to isolate genomic DNA from protoplasts. Mix gently the protoplast solution and transfer the duplicates of transfected protoplasts (2 × 1 mL) to one 5 mL tube. Wash the wells in which the protoplasts were incubated with 1 mL of water and transfer to the same 5 mL tube. Centrifuge for 15 min at 14 000 rpm. Discard the supernatant and add 800 μL of CTAB buffer preheated to 60°C and 3 μL of RNase (100 mg/mL). Vortex to dissolve the protoplasts pellet. Incubate for 1 h at 60°C in a thermomixer (mix by inverting every 15 min). Add 800 μL of chloroform:isoamyl alcohol solution (24:1), mix by inverting, incubate for 3 min at RT, and centrifuge for 10 min at 14 000 rpm. Transfer the clear, upper phase to a new 1.5 mL tube. Add 600 μL of cold (−20°C) isopropanol and incubate for 1 h at −20°C. Spin for 15 min at 14 000 rpm and 4°C. Discard the supernatant, add 1 mL of cold (−20°C) 70% ethanol and centrifuge for 5 min at 14 000 rpm and 4°C. Discard the supernatant, air dry and dilute in 20 μL TE buffer.

(19). Digest whole genomic DNA from the protoplasts after transfection with the relevant restriction enzyme that recognizes the Cas9 cutting site in a wild-type target sequence. New England BioLabs enzymes were used, and the restriction digestion reaction was prepared following the manufacturer’s protocol.

**NOTE:** If it is possible (i.e., if the restriction enzyme does not show the star activity (which means cleaving sequences which are similar, but not identical, to their defined recognition sequence) during extended digestion), perform the restriction digestion overnight.

(20). Perform PCR using Platinum Taq DNA Polymerase High Fidelity (Thermo Fisher Scientific) and following the manufacturer’s protocol. Add 5 μL of digested genomic DNA to 50 μL of PCR reaction containing the primers (step 8) in order to amplify part of the target gene with the gRNA sequence. Run the PCR reaction on the 1% agarose gel. The enrichment PCR reaction, after the restriction digestion of genomic DNA, amplifies exclusively part of the gene from edited protoplasts. Introduced mutations are resistant to restriction enzyme digestion due to the loss of the restriction site, so the PCR products should be derived only from the edited protoplasts.
(21). Cut out the band from the gel and clone into a pGEM-T vector. Transform *E. coli* with the recombinant vector to obtain single colonies carrying one gene copy.
  The enrichment PCR reaction is not completely effective, and many colonies may still carry plasmids without the mutation of the target sequence. To circumvent this problem, continue as follows.
(22). Before plasmid isolation and sequencing, prepare a colony PCR reaction to amplify part of the target gene, using a Platinum^TM^ Taq DNA Polymerase High Fidelity (Thermo Fisher Scientific) and following the manufacturer’s protocol. Touch the colony with a tip, transfer to the PCR mixture, mix by pipetting, and perform colony PCR. Digest the PCR product with the relevant restriction enzyme and run on the 1% agarose gel. If the PCR product cannot be restricted, it is likely that the target sequence is edited. Isolate plasmids from potentially edited colonies and sequence with M13F primer to determine the nature of the mutation.

**NOTE:** Some mutations may be present but do not lead to the loss of a restriction site. For example, if a single nucleotide is inserted at the border of the restriction site and shares the same identity as the nucleotide of the wild type sequence the restriction site will be unimpaired. This insertion cannot be detected with the PCR/RE assay but will cause a frameshift mutation and will lead to the production of a non-functional protein.

#### *Agrobacterium tumefaciens-*Mediated Transformation (Timing ∼18 Weeks)

*Agrobacterium*-mediated transformation is the longest part of targeted mutagenesis in *Brachypodium* species. It is advisable, therefore, to test first the efficacy of vectors using the transient protoplast assays described above to save time and resources. *Agrobacterium*-mediated *Brachypodium* transformation is effected by introducing the pOsCas9_sgRNA or pYPQ_Cas9_gRNA vectors to *A. tumefaciens* strain AGL1 using electroporation.

(23). Thaw on ice 40 μL of competent AGL1 cells (see **NOTE** for the *Agrobacterium* electrocompetent cells preparation). Add ∼500 ng of pOsCas9_sgRNA (from step 14) or pYPQ_Cas9_gRNA vector prepared according to the protocol by Lowder et al. (2015) and mix gently. Transfer the bacteria to an electroporation cuvette and apply one pulse of Agr program (capacitance 50 mF, load resistance 200 Ω, maximum power 25 W, current 25 mA and voltage 1800 V). Very slowly add 500 μL of LB medium to the cuvette and transfer the bacteria to a 1.5 mL tube and incubate for 3 h at 28°C with horizontal shaking at 125 rpm. Add 500 μL of LB medium to reduce bacterial density, mix gently by inverting the tube and then inoculate 50 μL of the bacterial culture on LB agar plates with spectinomycin at 50 μg/mL and rifampicin at 25 μg/mL for the pOsCas9_sgRNA vector, and on LB agar plates with kanamycin at 50 μg/mL and rifampicin at 25 μg/mL for the pYPQ_Cas9_gRNA vector. Culture upside down for 2 days at 28°C in the dark. From the many colonies produced, pick five and inoculate into 10 mL of LB + spectinomycin at 50 μg/mL and rifampicin at 25 μg/mL for the pOsCas9_sgRNA vector and LB + kanamycin at 50 μg/mL and rifampicin at 25 μg/mL for the pYPQ_Cas9_gRNA vector in a 50 mL plastic disposable tube. Grow for 2 days in the incubator shaker at 28°C with 250 rpm shaking in the dark and isolate plasmids using an Agrobacterium Plasmid Miniprep DNA Purification Kit according to the manufacturer’s instructions. Sequence vectors with the RB_F primer for the pOsCas9_sgRNA vector, or with the pTC14-F2 primer for the pYPQ_Cas9_gRNA vector (Supplementary Table S1). Prepare a glycerol stock of the *A. tumefaciens* with the pOsCas9_sgRNA or pYPQ_Cas9_gRNA by mixing the 500 μL of the 2 day-old liquid bacterial culture with 500 μL of 50% glycerol. Store at −80°C.

**NOTE:** To prepare *Agrobacterium* competent cells: (1) Refresh *Agrobacterium* in LB + rifampicin medium and grow at 28°C until you have nice single colonies (1–2 days); (2). Pick a single colony and inoculate in 10 mL LB + rifampicin. Grow at 28°C, shaking for two days; (3). Take 2 ml of the *Agrobacterium* culture and inoculate it into 100 mL of LB + rifampicin. Incubate at 28°C shaking until OD600 reaches 0.5; (4). Chill culture on ice for 10 min. Harvest cells by centrifugation (5000 rpm for 10 min at 4°C); (5). Wash pellet with 10 mL of cold HEPES pH 7 (filter sterilized). Do this gently. Spin down (5000 rpm for 10 min at 4°C). Repeat step above twice; (6). Wash pellet with 10 mL of cold 10% glycerol (filter sterilized). Spin; (7). Gently resuspend pellet in 1 mL of cold 10% glycerol; (8). Divide into 100 μL aliquots into chilled Eppendorf tubes and immediately freeze in liquid nitrogen. Store at −80°C.

(24). Collect green immature seeds from 7–9 week old plants. Remove the top glume from the seeds, and put ∼100 seeds into a 50 mL plastic disposable tube. Sterilize 2 min with 50 mL 70% ethanol, discard ethanol and add 50 mL 5% calcium hypochlorite, incubate for 10 min (mix gently by inverting), and rinse three times with sterile deionised water. Isolate immature embryos (Figure 3A) ∼0.3–0.7 mm in length and put them onto BdCIM medium. Cover the plates with Parafilm and incubate for 3 weeks at 25°C in the dark.

**NOTE:** Only very small immature embryos produce CEC at high frequency.

(25). After three weeks fragment the embryogenic masses (Figure 3B) into small pieces and transfer onto fresh BdCIM medium for another two weeks at 25°C in the dark. Discard the vitreous and friable parenchymatous cells.
(26). At week five, split CEC into 4–6 pieces and transfer them onto fresh BdCIM medium for another week at 25°C in the dark. Perform the transformation exactly after 6 weeks of callus induction.
(27). Three days before the planned transformation add 5 μL of *A. tumefaciens* from the glycerol stock (prepared in step 23) to 1 mL of LB + spectinomycin at 50 μg/mL, cover with aluminum foil and grow overnight at 28°C with 200 rpm shaking. Inoculate 200 μL of the overnight culture onto MGL + S50 + AS30 plates. Culture upside down for 2 days at 28°C in the dark.
(28). Scrape the *Agrobacterium* layer with a sterile scalpel and add to 10 mL BdAGM medium in a 50 mL plastic disposable tube. Shake vigorously and incubate for 45 min at 28°C with shaking at 220 rpm. Measure the optical density of the suspension at 600 nm and dilute to an OD600 between 0.9–1.0 with BdAGM medium.
(29). Transfer the CEC to a sterile plastic Petri dish, and cut them into the small pieces using a scalpel. Collect the callus in a 50 mL plastic disposable tube and cover with the *Agrobacterium* suspension. Incubate for 5 min (mixing by inverting), pipette out the bacterial suspension completely from the CEC and transfer them onto dry sterile filter paper in an empty Petri dish.

**NOTE:** Use three layers of filter paper in each Petri dish to ensure better CEC drying.

**NOTE:** Thorough drying of the CEC from bacterial suspension is extremely important.

Leave the open Petri dish with CEC under a laminar flow hood for seven min as a desiccation treatment. Transfer the CEC onto BdCCM medium, cover the plates with Parafilm and incubate for 2 days at 25°C in the dark.

(30). Transfer the CEC onto BdSM40 medium and culture for three weeks at 25°C in the dark.

**NOTE:** Do not transfer CEC overgrown with *Agrobacterium*.

(31). After three weeks split the transformed CEC (Figure 3C) into 4–6 pieces and transfer them onto BdSM30 medium for another three weeks at 25°C in the dark. Discard non-transformed dark brown calli.
(32). Six weeks after transformation, transfer the hygromycin-resistant calli onto BdRM medium and incubate for three weeks at 25°C under 16-h photoperiod.
(33). When shoots appear and reach a length not less than 2 cm, transfer them to plastic disposable tubes with BdGM medium. Culture at 25°C under a 16-photoperiod until the roots appear and the plants are at least a few cm long.

**NOTE:** Do not transfer smaller plants because they are very vulnerable and prone to dying.

(34). Transfer fully rooted plantlets to pots filled with soil mixed with vermiculite at a ratio of 3:1. Keep seedlings covered with a propagator lid during first two weeks. Grow plants in a greenhouse at 21 ± 1°C/16 h-photoperiod until the seeds are ready to collect. Collect the seeds and store at 4°C.

#### Analysis of Regenerated Plants (Timing ∼1 Week)

Analyze the regenerated plants for T-DNA insertion and mutation in the target sequence.

(35). Isolate the DNA from the leaves of regenerated plants using a quick protocol. Carry out the whole procedure at RT. Cut a piece of a young leaf around 1 cm^2^ and macerate using a plastic pestle in a 1.5 mL tube. Add 400 μL of extraction buffer and continue to grind the leaf until a homogenous solution is attained. Centrifuge for 3 min at 14 000 rpm, and transfer 300 μL of the supernatant to a new 1.5 mL tube. Add 300 μL of isopropanol, vortex briefly and centrifuge for 5 min at 14 000 rpm. Discard the supernatant and wash the pellet with 300 μL 70% ethanol. Centrifuge for 5 min at 14 000 rpm again, discard the supernatant and dry the pellet. Resuspend the pellet in 50 μL TE buffer. Use 1 μL of isolated DNA for 20 μL of PCR reaction.
(36). Assay the transformation of regenerated plants using one of the following options (A or B).

**(A) Determination of the presence or absence of the Cas9 gene**

Use the primers that amplify part of the gene encoding the Cas9 protein in pOsCas9_sgRNA or pYPQ_Cas9_gRNA vectors (Supplementary Table S1). Use the Color Taq PCR Master Mix for the Cas9 PCR reaction (see section “Reagent Setup”) and run using following program: 94°C for 3 min; (94°C for 30 s; 58°C for 30 s; 72°C for 30 s) – 30 cycles; 72°C for 7 min; 4°C for ∞. Run the PCR product on a 1% agarose gel. A 382-nucleotide PCR product for the pOsCas9_sgRNA vector and a 357-nucleotide PCR product for pYPQ_Cas9_gRNA indicate successful transformation. Always include a negative control – DNA isolated from the wild-type plant, and a positive control – for example vector DNA (pOsCas9_sgRNA or pYPQ_Cas9_gRNA).

**(B) Determination of the presence or absence of the gene conferring resistance to hygromycin (HygR test)**

Snip 1 – 1.5 cm from the tips of the youngest, but completely expanded leaves of each regenerated plant. Place the leaf immediately in BdHygR medium with its abaxial side down and slightly angled (Figure 3E). Do not damage the leaf and make sure the cut tip of the leaf does not go all the way through the media. 1 leaf/cm^2^ is the appropriate density. Cover the plates with Parafilm and culture for 5 days at 25°C under a 16-photoperiod. Hygromycin resistant leaves remain green, whereas susceptible leaves turn white at the cut edge.

To determine the success of editing, follow the PCR/RE assay, cloning and sequencing (steps 37 and 38). Alternatively, skip step 37 and go directly to step 38, as we found that some of the mutations cannot be detected using the PCR/RE assay, and yet most of the transformed plants are edited (see section “RESULTS AND DISCUSSION”).

(37). Amplify part of the targeted gene using the primers designed in step 8 and Platinum^TM^ Taq DNA Polymerase High Fidelity (Thermo Fisher Scientific). Prepare a 25 μL reaction according to the manufacturer’s protocol. Restrict 15 μL of the PCR product with the relevant restriction enzyme according to the manufacturer’s protocol. As a control, a wild-type plant should be included in the analysis. Run the digested PCR product on a 1% agarose gel.

Three outcomes are possible:

• One band of a size corresponding to the part of the gene defined by the primers. This suggests that the restriction site was lost by mutation of all copies of the target gene.
• Two bands that reflect two fragments of the amplified sequence following digestion by the restriction enzyme. This suggests that restriction sites are still present in the target sequence, and that no copies of the target gene have been edited, or an induced mutation did not lead to the loss of a restriction site.
• Three bands suggest that at least one copy of the gene has been successfully edited. The other copies may not be edited or the induced mutation did not lead to the loss of a restriction site.

**NOTE:** Another possibility for the results of three bands is that the target band was not fully digested due to the incomplete effectiveness of the restriction enzyme activity. Thus, it is necessary to include for each experiment the control reaction with the DNA from wild-type plant.

Select for further analysis only those plants that have at least one edited copy of the gene. Do not include plants from which the PCR product has been completely digested by the restriction enzyme.

**NOTE:** Alternatively, in *B. distachyon*, the amplified PCR product could be analyzed with the use of T7 Endonuclease 1 (T7E1, New England Biolabs) instead of the digestion using the restriction enzyme. However, this enzyme also has its limitations and may not detect some mutations. For example, it works best at C mismatches and does not recognize all DNA mismatches.

(38). Clone the undigested PCR product into a pGEM-T vector in order to determine the nature of the mutations. Non-digested PCR product is used, in order to include all gene copies, regardless of whether they are edited or not. If the outcome was one band, this band can be directly isolated from the gel and used for cloning. If three bands were observed, run the rest of undigested PCR product (10 μL) on a 1% agarose, isolate from the gel and use for cloning. Transform *E. coli* with the recombinant vector, isolate plasmids from individual bacterial colonies, and sequence using a M13For primer. Align the sequence with that of wild-type.

**NOTE:** Since each individual colony carries only one copy of the gene, five colonies should be sequenced in order to be sure of including both gene copies in *B. distachyon* and 10 colonies to include all gene copies in *B. hybridum*.

If the T1 or subsequent generations are used for phenotypic analysis, it is best to select those plants in which the T-DNA cassette has been lost by segregation.

(39). The presence or absence of the T-DNA cassette is determined by using one of the methods presented in step 36 – PCR reaction (option A) or HygR test (option B).

## Results and Discussion

**FIGURE 4:**
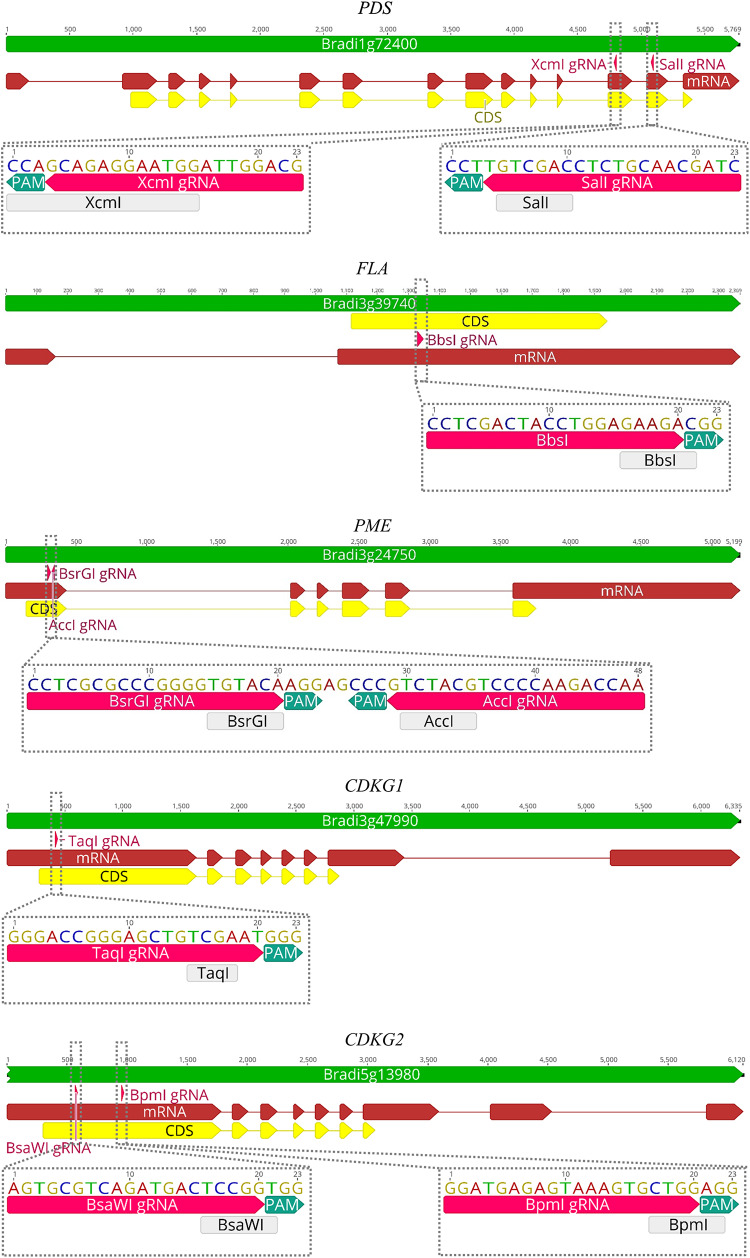
Maps of *PDS*, *FLA*, *PME*, *CDKG1*, and *CDKG2* genes with marked positions of gRNAs (pink arrows) and appropriate restriction sites (light gray rectangles). PAM – protospacer-adjacent motif.

**FIGURE 5:**
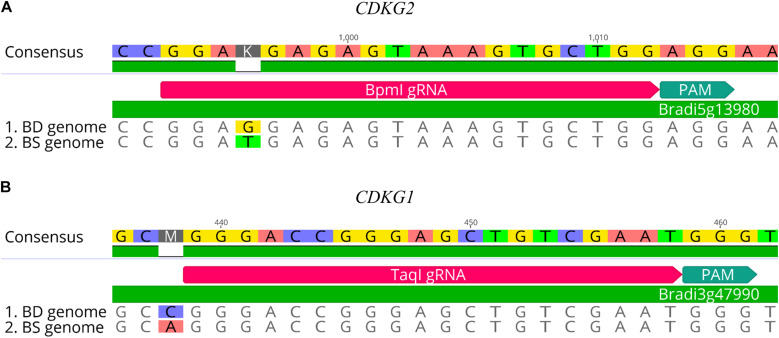
Examples of gRNA sequences designed for targeted mutagenesis in *B. hybridum.* **(A)** SNP in the target sequence of Bd and Bs genomes located distal to the PAM. The gRNA was designed from the Bs genome sequence. **(B)** Example of SNP differentiating Bd and Bs genomes occurring outside the target 20-nucleotide sequence.

**FIGURE 6:**
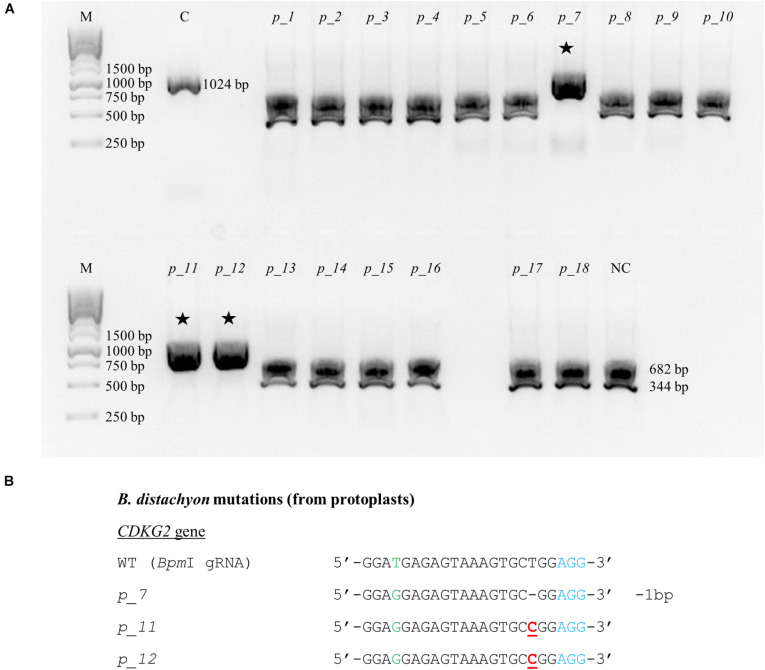
Example of the nature of mutations obtained using CRISPR/Cas9 targeted mutagenesis with *Bpm*I gRNA in Bd21 protoplasts. **(A)** Analysis of the obtained colonies using PCR/RE assay and gel electrophoresis. Lane M, molecular weight marker, lane C, control – undigested PCR product, lanes *p_1* – *p_18*, analyzed colonies obtained during the transient protoplast assay, lane NC, negative control – the PCR product from the WT plant digested using *Bpm*I restriction enzyme. bp – base pairs. Edited sequences are indicated by asterisks. **(B)** Results of sequencing of plasmids from individual colonies. Deletions are denoted as hyphens (-) and substitutions as bold, underlined red letters. The PAM sequence is shown in blue. SNP in the target sequence of Bd and Bs genomes is shown in green. bp – base pairs.

CRISPR/Cas9 technology enables rapid and efficient targeted mutagenesis. We targeted five different genes (*PDS*, *FLA*, *PME*, *CDKG1* and *CDKG2*) in a model grass species, diploid *B. distachyon*, and two of them (*CDKG1* and *CDKG2*) in its allotetraploid derivative *B. hybridum*. Single gRNA sequences were designed for *FLA* gene (*Bbs*I gRNA) and *CDKG1* gene (*Taq*I gRNA) and two gRNA sequences for *PDS* gene (*Xcm*I gRNA, *Sal*I gRNA), *PME* gene (*Bsr*GI gRNA, *Acc*I gRNA) and *CDKG2* gene (*Bpm*I gRNA and *Bsa*WI gRNA) (Figure 4). The efficiency of all designed gRNAs in *Brachypodium* genome editing was tested using a transient protoplast assay. All the gRNA sequences targeted the conserved regions of the genes in both the Bd and Bs genome, with the exception that *Bpm*I gRNA contains one single nucleotide polymorphism (SNP) distal to PAM (Figure 5A). The SNP in the region outside the gRNA sequence helps to differentiate between all of the gene copies from the Bd and Bs genomes (Figure 5B). The *Bpm*I gRNA is complementary to the Bs *CDKG2* sequence. The transient protoplast assay was conducted on protoplasts of Bd21 leaves and, since the SNP is in the “non-seed” gRNA region, the Cas9 endonuclease was able to introduce DSBs in the target gene. Two different mutations were obtained: deletion of T and substitution of T by C (Figure 6). It confirmed that *Bpm*I gRNA, despite the SNP, is functional in editing of Bd *CDKG2* copy. Positive results of the transient protoplast assay were also obtained for *Bsr*GI gRNA (the obtained mutation: deletion of T or G). Although no mutations were detected by the transient protoplast assay for the remaining gRNAs, they proved nevertheless to be effective in editing the *Brachypodium* genome, as demonstrated in the mutants described below. False negative results may arise because some of the mutations do not lead to a loss of the restriction site. On the other hand, depending on the activity of individual restriction enzymes, not all unedited sequences may be successfully digested. Therefore, when analyzing individual colonies, a large number of them may have a copy of the gene that has not been edited. Increasing the number of colonies analyzed, increases the likelihood of detecting mutations in protoplasts. Summarizing the results of the transient protoplast assay, we suggest, that the use of transient protoplast assay is optional and can be omitted, but due to its relatively low cost and speed, it might be useful for selecting the best gRNA among several designed for one gene, in order to save time and resources during the long process of *Agrobacterium*-mediated transformation. Moreover, as the transient protoplast assay can also be used for other purposes than just analysis of gRNA efficiency, for example for study the subcellular protein localisation (Jung et al., 2015), it seems reasonable to include our optimized, shortened protocol here.

**FIGURE 7:**
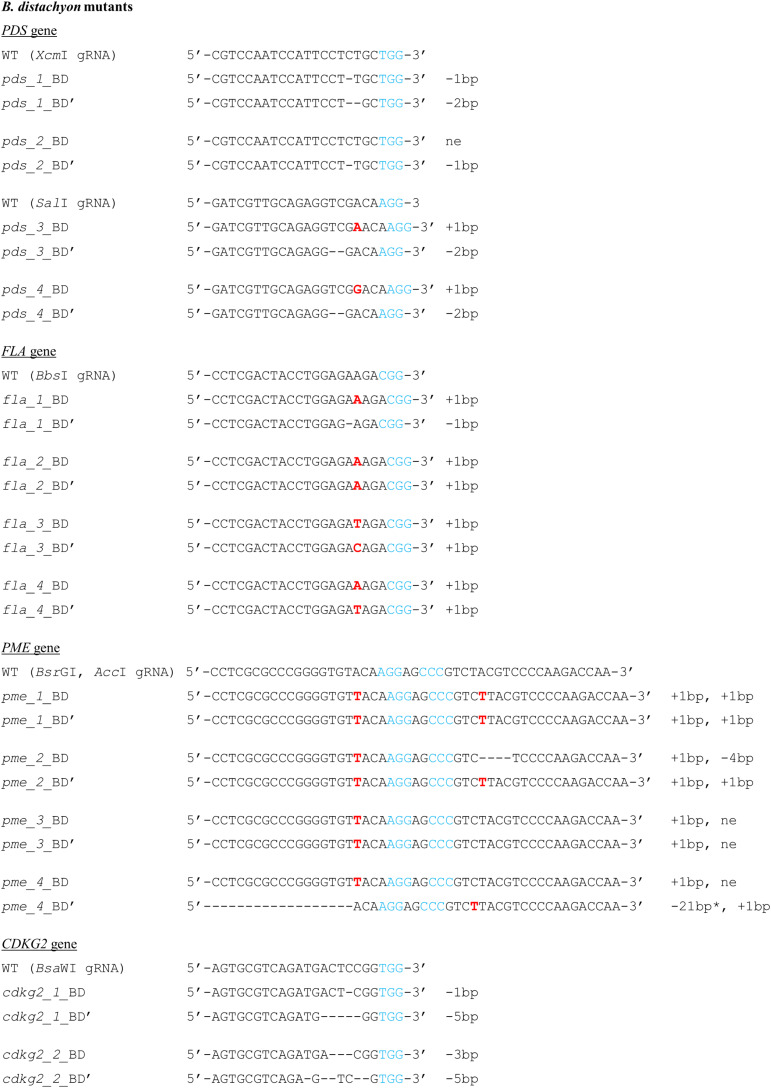
Nature of mutations obtained using CRISPR/Cas9 targeted mutagenesis in T0 plants of *B. distachyon*. Deletions are denoted as hyphens (-) and insertions as bold red letters. The PAM sequence is shown in blue. Individual plants are marked with numbers. The sequences of both alleles are presented and marked as BD, BD’. * - the mutation goes beyond the gRNA sequence. bp – base pairs, ne – not edited sequence.

**FIGURE 8:**
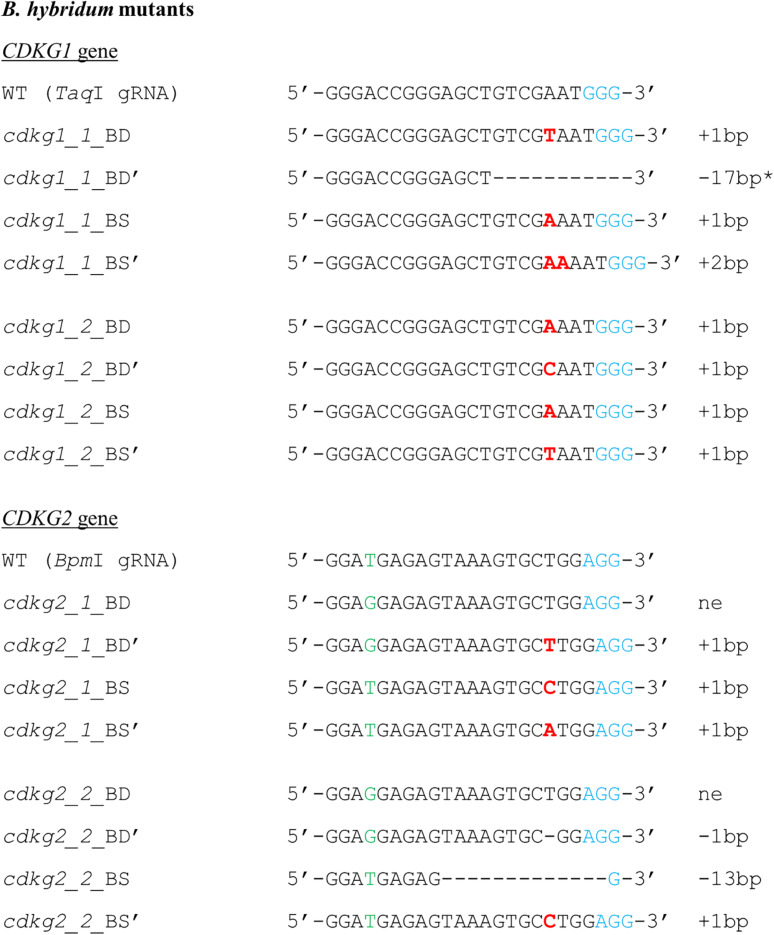
Nature of mutations obtained using CRISPR/Cas9 targeted mutagenesis in T0 plants of *B. hybridum*. Deletions are denoted as hyphens (-) and insertions as bold red letters. The PAM sequence is shown in blue. Individual plants are marked with numbers. The sequences of both alleles are presented and marked as BD, BD’ or BS, BS’. SNP in the target sequence of Bd and Bs genomes is shown in green. * - the mutation goes beyond the gRNA sequence. bp – base pairs, ne – not edited sequence.

All of the plants regenerated after *Agrobacterium*-mediated transformation were assayed for transformation status, and if positive, followed by assays for mutations. Twenty-two out of 24 transformed plants were successfully edited (Table 1). Figure 7 presents the nature of the mutations of *B. distachyon*, and Figure 8 shows those in *B. hybridum*. Some of the mutants obtained had exactly the same mutations, so they are shown only once in the figure. Our results show that Cas9 activity results mainly in small indels three nucleotides upstream of the PAM (Figures 7, 8), which caused frameshift mutations. Single nucleotide insertions or deletions have been reported previously in other *B. distachyon* mutants (O’Connor et al., 2017; Raissig et al., 2017; Gao et al., 2019). We also observed larger deletions at the target sequence, which involved 21 bp, 17 bp or 13 bp (Figures 7, 8).

**TABLE 1:** List of all obtained transgenic lines.

	**Transgenic line name**	**Species/Genotype**	**Target gene**	**Editing status**
1	*Bd21_pds_l*	*B. distachyon*/Bd21	*PDS*	Edited
2	*Bd21_pds_2*	*B. distachyon*/Bd21	*PDS*	Edited
3	*Bd21_pds_3*	*B. distachyon*/Bd21	*PDS*	Edited
4	*Bd21_pds_4*	*B. distachyon*/Bd21	*PDS*	Edited
5	*Bd21_fla_l*	*B. distachyon*/Bd21	*FLA*	Edited
6	*Bd21_fla_2*	*B. distachyon*/Bd21	*FLA*	Edited
7	*Bd21_fla_3*	*B. distachyon*/Bd21	*FLA*	Edited
8	*Bd21_fla_4*	*B. distachyon*/Bd21	*FLA*	Edited
9	Bd21_fla_4*	*B. distachyon*/Bd21	*FLA*	Edited
10	*Bd21_FLA_l*	*B. distachyon*/Bd21	*FLA*	Not edited
11	*Bd21_FLA_2*	*B. distachyon*/Bd21	*FLA*	Not edited
12	*Bd21_pme_l*	*B. distachyon*/Bd21	*PME*	Edited
13	*Bd21_pme_2*	*B. distachyon*/Bd21	*PME*	Edited
14	*Bd21_pme_3*	*B. distachyon*/Bd21	*PME*	Edited
15	Bd21_pme_3*	*B. distachyon*/Bd21	*PME*	Edited
16	*Bd21_pme_4*	*B. distachyon*/Bd21	*PME*	Edited
17	*Bd21_cdkg2_l*	*B. distachyon*/Bd21	*CDKG2*	Edited
18	Bd21_cdkg2_l*	*B. distachyon*/Bd21	*CDKG2*	Edited
19	*Bd21_cdkg2_2*	*B. distachyon*/Bd21	*CDKG2*	Edited
20	Bd21_cdkg2_2*	*B. distachyon*/Bd21	*CDKG2*	Edited
21	*ABR113_cdkgl_l*	*B. hybridum*/ABR113	*CDKG1*	Edited
22	*ABR113_cdkgl_2*	*B. hybridum*/ABR113	*CDKG1*	Edited
23	*ABR113_cdkg2_l*	*B. hybridum*/ABR113	*CDKG2*	Edited
24	*ABR113_cdkg2_2*	*B. hybridum*/ABR113	*CDKG2*	Edited

*The plants with the same name and differing only by the asterisk had exactly the same kind of mutation.*

We obtained four different *pds* mutants (Figure 7), of which three were edited in both gene copies (biallelic mutants) and showed the expected albino phenotype (Figure 3F). One of the *PDS* mutants had just one copy of the gene edited (single-allelic mutant) and resembled a wild-type plant, indicating that all copies of the gene must be knocked-out to change the phenotype. Similar results were reported previously by Shan et al. (2013) and Pan et al. (2016).

Four different *fla* biallelic mutants were obtained (Figure 7). *PME* was the only gene we targeted simultaneously with two different gRNAs. We expected, that larger deletions, covering the area between both gRNAs, will occur if the DSBs are simultaneously induced at both gRNA sites. We produced four different *pme* mutants (Figure 7) and observed that *Bsr*GI gRNA edited both gene copies in all four, whereas *Acc*I gRNA edited both gene copies in only two of the four. This confirmed the better efficiency of the *Bsr*GI gRNA than *Acc*I gRNA in *Brachypodium* editing and may be in part explained by different cleavage efficiencies of various gRNAs (Bruegmann et al., 2019). It is also consistent with the results obtained using the transient protoplast assay, as positive results were obtained for *Bsr*GI gRNA and not for *Acc*I gRNA. Even if both gRNAs worked in the same plant, we did not observe bigger deletions in the region between the gRNAs; instead we observed small lesions at both gRNA target sites. It seems, therefore, that the use of vectors with two different gRNA sequences targeting the same gene increases the likelihood of mutation. Such vectors could also be useful for targeted mutagenesis of two different genes at the same time.

We also targeted the *CDKG1* and *CDKG2* genes in both *B. distachyon* and *B. hybridum.* We obtained two different *cdkg2* biallelic mutants of *B. distachyon* (Figure 7), and *B. hybridum cdkg1* mutants and *B. hybridum cdkg2* mutants (Figure 8). *B. hybridum cdkg1* mutants carry the mutation in all of their gene copies, whereas the *cdkg2* mutants of *B. hybridum* carried the mutations in three out of four gene copies. As shown by the *pds* mutants, an altered plant phenotype requires all of the gene copies to be knocked-out. Thus, from the T1 generation plants of *cdkg2 B. hybridum* mutants we selected those with four mutated alleles using the PCR/RE assay as well as cloning and sequencing. As the *cdkg1* as well as *cdkg2* mutants were obtained, our next step will be the simultaneous mutagenesis of both genes in the same individual. To the best of our knowledge, this is the first example of the successful use of CRISPR/Cas9 technology in targeted mutagenesis of the allotetraploid species *B. hybridum*. Targeted mutagenesis such as TALENs and CRISPR/Cas9 have been used to edit multiple alleles in another allopolyploid species – allohexaploid wheat (Wang et al., 2014; Singh et al., 2018).

Although sequence analysis provides the definitive identification of lesions, it is useful to design a rapid screening method. We presented two different methods for assaying the success transformation of regenerated plants. The method based on PCR is commonly used in many laboratories, and is very sensitive and accurate. However, as an alternative we presented the HygR test, which is fast and allows the simultaneous analysis of many regenerated plants with little effort and low cost.

For the analysis of transformed plants in terms of mutation, we proposed the PCR/RE assay and method based on cloning and sequencing. It should be noted that one of the three mutations in both of the *B. hybridum cdkg2* mutants was the addition of a C nucleotide (Figure 8) which did not lead to the loss of a *Bpm*I restriction site (CTGGAG) and could not be detected by a PCR/RE assay. Nevertheless, the remaining mutations were resistant to restriction digestion and implied the occurrence of mutation and the need for further analysis of all gene copies using cloning and sequencing. The probability is low that the mutations undetectable using a PCR/RE assay will occur in all copies of the gene, thus a PCR/RE assay might be useful for the screening of a large number of transformed plants. As we mentioned before, most of our transformed plants were edited, so it may also be feasible to skip the PCR/RE analysis and perform the cloning and sequencing directly. A T7E1 assay (Xie and Yang, 2013) is an alternative method for detecting mutation and can be especially useful if it is difficult to design the gRNA sequence with a restriction site at the Cas9 cutting site. The T7EI assay is based on a PCR reaction, followed by amplicon denaturation and subsequent reannealing. If a mutation occurs at the target site, heteroduplexes are formed between the mutant and wild type sequences. Heteroduplexes can be detected by cleaving with the T7EI enzyme. This method might be an alternative way for detecting of mutations in *B. distachyon*. However, applying this method for the detection of mutations in *B. hybridum* is fraught with difficulty, as the Bd and Bs homoeologues are not completely identical. Another method has been proposed recently to analyze mutations identified by a transient protoplast assay, in which double-stranded oligodeoxynucleotide (dsODN) is introduced into the Cas9-induced double stranded break, and PCR with dsODN-specific primers and target gene-specific primers is performed (Nadakuduti et al., 2019). However, the greatest disadvantage of this method is that the addition of dsODNs to the transfection reaction can substantially suppress the frequency of mutation. Taking all of this into account, we recommend analysis based upon restriction enzyme digestion, cloning and sequencing for the detection of mutations both in protoplasts and regenerated plants.

## Conclusion

Precise, targeted knock-out by gene editing of diploid *B. distachyon* and allopolyploid *B. hybridum* has been demonstrated using the protocol presented. The method should, therefore, prove useful in functional genomic studies in these model grasses.

## Data Availability Statement

All datasets generated for this study are included in the article/supplementary material.

## Author Contributions

AB, EG, KJ, CN, JD, and RH conceived the experiments. KH, AB, AP, MR-J, and MG conducted the study and processed the data. KH and AB wrote the manuscript. EG, CN, MG, KJ, GJ, JD and RH discussed the results and contributed to manuscript writing. All authors have read and approved the final manuscript.

## Conflict of Interest

The authors declare that the research was conducted in the absence of any commercial or financial relationships that could be construed as a potential conflict of interest.

## Footnotes

1 http://www.rgenome.net/cas-offinder/

## References

[B1] Al-Kaff N. Knight E. Bertin I. Foote T. Hart N. Griffiths S. (2008). Detailed dissection of the chromosomal region containing the Ph1 locus in wheat *Triticum aestivum*: with deletion mutants and expression profiling. *Ann. Bot.* 101 863–872. 10.1093/aob/mcm252 17951583 PMC2710213

[B2] Alves S. C. Worland B. Thole V. Snape J. W. Bevan M. W. Vain P. (2009). A protocol for *Agrobacterium*-mediated transformation of *Brachypodium distachyon* community standard line Bd21. *Nat. Protoc.* 4 638–649. 10.1038/nprot.2009.30 19360019

[B3] Bae S. Park J. Kim J. S. (2014). Cas-OFFinder: a fast and versatile algorithm that searches for potential off-target sites of Cas9 RNA-guided endonucleases. *Bioinformatics* 30 1473–1475. 10.1093/bioinformatics/btu048 24463181 PMC4016707

[B4] Bragg J. N. Wu J. Gordon S. P. Guttman M. E. Thilmony R. Lazo G. R. (2012). Generation and characterization of the Western Regional Research Center *Brachypodium* T-DNA insertional mutant collection. *PLoS One* 7:e41916. 10.1371/journal.pone.0041916 23028431 PMC3444500

[B5] Bruegmann T. Deecke K. Fladung M. (2019). Evaluating the efficiency of gRNAs in CRISPR/Cas9 mediated genome editing in poplars. *Int. J. Mol. Sci.* 20:E3623. 10.3390/ijms20153623 31344908 PMC6696231

[B6] Caroll D. (2008). Progress and prospects: zinc-finger nucleasesas gene therapy agents. *Gene Ther.* 15 1463–1468. 10.1038/gt.2008.145 18784746 PMC2747807

[B7] Chen Y. Fokar M. Kang M. Chen N. Allen R. D. Chen Y. (2018). Phosphorylation of *Arabidopsis* SINA2 by CDKG1 affects its ubiquitin ligase activity. *BMC Plant Biol.* 18:147. 10.1186/s12870-018-1364-8 30012094 PMC6048857

[B8] Christian M. Cermak T. Doyle E. L. Schmidt C. Zhang F. Hummel A. (2010). Targeting DNA double-strand breaks with TAL effector nucleases. *Genetics* 186 757–761. 10.1534/genetics.110.120717 20660643 PMC2942870

[B9] Christiansen P. Andersen C. H. Didion T. Folling M. Nielsen K. K. (2005). A rapid and efficient transformation protocol for the grass *Brachypodium distachyon*. *Plant Cell Rep.* 23 751–758. 10.1007/s00299-004-0889-5 15503032

[B10] Cong L. Ran F. A. Cox D. Lin S. Barretto R. Habib N. (2013). Multiplex genome engineering using CRISPR/Cas systems. *Science* 339 819–823. 10.1126/science.1231143 23287718 PMC3795411

[B11] Draper J. Mur L. A. Jenkins G. Ghosh-Biswas G. C. Bablak P. Hasterok R. (2001). *Brachypodium distachyon* a new model system for functional genomics in grasses. *Plant Physiol.* 127 1539–1555. 11743099 PMC133562

[B12] Gao M. Geng F. Klose C. Staudt A.-M. Huang H. Nguyen D. (2019). Phytochromes measure photoperiod in *Brachypodium*. *bioRxiv* [Preprint]. 10.1101/697169

[B13] Hsia M. M. O’malley R. Cartwright A. Nieu R. Gordon S. P. Kelly S. (2017). Sequencing and functional validation of the JGI *Brachypodium distachyon* T-DNA collection. *Plant J.* 91 361–370. 10.1111/tpj.13582 28432803

[B14] Jiang W. Zhou H. Bi H. Fromm M. Yang B. Weeks D. P. (2013). Demonstration of CRISPR/Cas9/sgRNA-mediated targeted gene modification in *Arabidopsis*, tobacco, sorghum and rice. *Nucleic Acids Res.* 41:e188. 10.1093/nar/gkt780 23999092 PMC3814374

[B15] Jinek M. Chylinski K. Fonfara I. Hauer M. Doudna J. A. Charpentier E. (2012). A programmable dual-RNA-guided DNA endonuclease in adaptive bacterial immunity. *Science* 337 816–821. 10.1126/science.1225829 22745249 PMC6286148

[B16] Jung H. Yan J. Zhai Z. Vatamaniuk O. K. (2015). “Gene functional analysis using protoplast transient assays,” in *Plant Functional Genomics: Methods in Molecular Biology*, eds Alonso J. Stepanova A. (New York, NY: Humana Press). 10.1007/978-1-4939-2444-8_22 25757786

[B17] Kaur N. Alok A. Shivani K. N. Pandey P. Awasthi P. Tiwari S. (2018). CRISPR/Cas9-mediated efficient editing in phytoene desaturase (PDS) demonstrates precise manipulation in banana cv, Rasthali genome. *Funct. Integr. Genomics* 18 89–99. 10.1007/s10142-017-0577-5 29188477

[B18] Li T. Liu B. Spalding M. H. Weeks D. P. Yang B. (2012). High-efficiency TALEN-based gene editing produces disease-resistant rice. *Nat. Biotechnol.* 30 390–392. 10.1038/nbt.2199 22565958

[B19] Lowder L. G. Zhang D. Baltes N. J. Paul J. W. III Tang X. Zheng X. (2015). A CRISPR/Cas9 toolbox for multiplexed plant genome editing and transcriptional regulation. *Plant Physiol.* 169 971–985. 10.1104/pp.15.00636 26297141 PMC4587453

[B20] Ma X. Qiao Z. Chen D. Yang W. Zhou R. Zhang W. (2015). CYCLIN-DEPENDENT KINASE G2 regulates salinity stress response and salt mediated flowering in *Arabidopsis thaliana*. *Plant Mol. Biol.* 88 287–299. 10.1007/s11103-015-0324-z 25948280

[B21] Miao J. Guo D. Zhang J. Huang Q. Qin G. Zhang X. (2013). Targeted mutagenesis in rice using CRISPR-Cas system. *Cell Res.* 23 1233–1236. 10.1038/cr.2013.123 23999856 PMC3790239

[B22] Miller J. C. Holmes M. C. Wang J. Guschin D. Y. Lee Y. L. Rupniewski I. (2007). An improved zinc-finger nuclease architecture for highly specific genome editing. *Nat. Biotechnol.* 25 778–785. 10.1038/nbt1319 17603475

[B23] Miller J. C. Tan S. Qiao G. Barlow K. A. Wang J. Xia D. F. (2011). A TALE nuclease architecture for efficient genome editing. *Nat. Biotechnol.* 29 143–148. 10.1038/nbt.1755 21179091

[B24] Nadakuduti S. S. Starker C. G. Ko D. K. Jayakody T. B. Buell C. R. Voytas D. F. (2019). Evaluation of methods to assess in vivo activity of engineered genome-editing nucleases in protoplasts. *Front. Plant Sci.* 10:110. 10.3389/fpls.2019.00110 30800139 PMC6376315

[B25] O’Connor D. L. Elton S. Ticchiarelli F. Hsia M. M. Vogel J. P. Leyser O. (2017). Cross-species functional diversity within the PIN auxin efflux protein family. *Elife* 6:e31804. 10.7554/eLife.31804 29064367 PMC5655145

[B26] Pacurar D. I. Thordal-Christensen H. Nielsen K. K. Lenk I. (2008). A high-throughput *Agrobacterium*-mediated transformation system for the grass model species *Brachypodium distachyon* L. *Transgenic Res.* 17 965–975. 10.1007/s11248-007-9159-y 18064538

[B27] Pan C. Ye L. Qin L. Liu X. He Y. Wang J. (2016). CRISPR/Cas9-mediated efficient and heritable targeted mutagenesis in tomato plants in the first and later generations. *Sci. Rep.* 6:24765. 10.1038/srep46916 27097775 PMC4838866

[B28] Pinski A. Betekhtin A. Sala K. Godel-Jedrychowska K. Kurczynska E. Hasterok R. (2019). Hydroxyproline-rich glycoproteins as markers of temperature stress in the leaves of *Brachypodium distachyon*. *Int. J. Mol. Sci.* 20:E2571. 10.3390/ijms20102571 31130622 PMC6567261

[B29] Qin Z. Bai Y. Muhammad S. Wu X. Deng P. Wu J. (2019). Divergent roles of FT-like 9 in flowering transition under different day lengths in *Brachypodium distachyon*. *Nat. Commun.* 10:812. 10.1038/s41467-019-08785-y 30778068 PMC6379408

[B30] Raissig M. T. Matos J. L. Anleu Gil M. X. Kornfeld A. Bettadapur A. Abrash E. (2017). Mobile MUTE specifies subsidiary cells to build physiologically improved grass stomata. *Science* 355 1215–1218. 10.1126/science.aan3164 28302860

[B31] Sander J. D. Dahlborg E. J. Goodwin M. J. Cade L. Zhang F. Cifuentes D. (2011). Selection-free zinc-finger-nuclease engineering by context-dependent assembly (CoDA). *Nat. Methods* 8 67–69. 10.1038/nmeth.1542 21151135 PMC3018472

[B32] Shan Q. Wang Y. Li J. Gao C. (2014). Genome editing in rice and wheat using the CRISPR/Cas system. *Nat. Protoc.* 9 2395–2410. 10.1038/nprot.2014.157 25232936

[B33] Shan Q. Wanga Y. Chena K. Lianga Z. Lia J. Zhanga Y. (2013). Rapid and efficient gene modification in rice and *Brachypodium* using TALENs. *Mol. Plant* 6 1365–1368. 10.1093/mp/sss162 23288864 PMC3968307

[B34] Shukla V. K. Doyon Y. Miller J. C. Dekelver R. C. Moehle E. Worden S. E. (2009). Precise genome modification in the crop species Zea mays using zinc-finger nucleases. *Nature* 459 437–441. 10.1038/nature07992 19404259

[B35] Singh M. Kumar M. Albertsen M. C. Young J. K. Cigan A. M. (2018). Concurrent modifications in the three homeologs of Ms45 gene with CRISPR-Cas9 lead to rapid generation of male sterile bread wheat (*Triticum aestivum* L.). *Plant Mol. Biol.* 97 371–383. 10.1007/s11103-018-0749-2 29959585

[B36] Tian S. Jiang L. Gao Q. Zhang J. Zong M. Zhang H. (2017). Efficient CRISPR/Cas9-based gene knockout in watermelon. *Plant Cell Rep.* 36 399–406. 10.1007/s00299-016-2089-5 27995308

[B37] Townsend J. A. Wright D. A. Winfrey R. J. Fu F. Maeder M. L. Joung J. K. (2009). High-frequency modification of plant genes using engineered zinc-finger nucleases. *Nature* 459 442–445. 10.1038/nature07845 19404258 PMC2743854

[B38] Upadhyay S. K. Kumar J. Alok A. Tuli R. (2013). RNA-guided genome editing for target gene mutations in wheat. *G3 (Bethesda)* 3 2233–2238. 10.1534/g3.113.008847 24122057 PMC3852385

[B39] Vain P. Worland B. Thole V. Mckenzie N. Alves S. C. Opanowicz M. (2008). *Agrobacterium*-mediated transformation of the temperate grass *Brachypodium distachyon* (genotype Bd21) for T-DNA insertional mutagenesis. *Plant Biotechnol. J.* 6 236–245. 10.1111/j.1467-7652.2007.00308.x 18004984

[B40] van der Schuren A. Voiniciuc C. Bragg J. Ljung K. Vogel J. Pauly M. (2018). Broad spectrum developmental role of *Brachypodium* AUX1. *New Phytol.* 219 1216–1223. 10.1111/nph.15332 29949662 PMC6100110

[B41] Vogel J. Hill T. (2008). High-efficiency *Agrobacterium*-mediated transformation of *Brachypodium distachyon* inbred line Bd21-3. *Plant Cell Rep.* 27 471–478. 10.1007/s00299-007-0472-y 17999063

[B42] Wang Y. Cheng X. Shan Q. Zhang Y. Liu J. Gao C. (2014). Simultaneous editing of three homoeoalleles in hexaploid bread wheat confers heritable resistance to powdery mildew. *Nat. Biotechnol.* 32 947–951. 10.1038/nbt.2969 25038773

[B43] Wendt T. Holm P. B. Starker C. G. Christian M. Voytas D. F. Brinch-Pedersen H. (2013). TAL effector nucleases induce mutations at a pre-selected location in the genome of primary barley transformants. *Plant Mol. Biol.* 83 279–285. 10.1007/s11103-013-0078-4 23689819 PMC7880306

[B44] Xie K. Yang Y. (2013). RNA-guided genome editing in plants using a CRISPR-Cas system. *Mol. Plant* 6 1975–1983. 10.1093/mp/sst119 23956122

[B45] Yan J. He H. Fang L. Zhang A. (2018). Pectin methylesterase31 positively regulates salt stress tolerance in Arabidopsis. *Biochem. Biophys. Res. Commun.* 496 497–501. 10.1016/j.bbrc.2018.01.025 29307824

[B46] Zhang F. Maeder M. L. Unger-Wallace E. Hoshaw J. P. Reyon D. Christian M. (2010). High frequency targeted mutagenesis in *Arabidopsis thaliana* using zinc finger nucleases. *Proc. Natl. Acad. Sci. U.S.A.* 107 12028–12033. 10.1073/pnas.0914991107 20508152 PMC2900673

[B47] Zhang Y. Zhang F. Li X. Baller J. A. Qi Y. Starker C. G. (2013). Transcription activator-like effector nucleases enable efficient plant genome engineering. *Plant Physiol.* 161 20–27. 10.1104/pp.112.205179 23124327 PMC3532252

[B48] Zheng T. Nibau C. Phillips D. W. Jenkins G. Armstrong S. J. Doonan J. H. (2014). CDKG1 protein kinase is essential for synapsis and male meiosis at high ambient temperature in *Arabidopsis thaliana*. *Proc. Natl. Acad. Sci. U.S.A.* 111 2182–2187. 10.1073/pnas.1318460111 24469829 PMC3926089

