# Supplementary figures and images for: A CRISPR/Cas9-Based Mutagenesis Protocol for Brachypodium distachyon and Its Allopolyploid Relative, Brachypodium hybridum
Source: Front Plant Sci. 2020 May 20;11:614. doi: 10.3389/fpls.2020.00614 (PMC7251944; doi:10.3389/fpls.2020.00614)

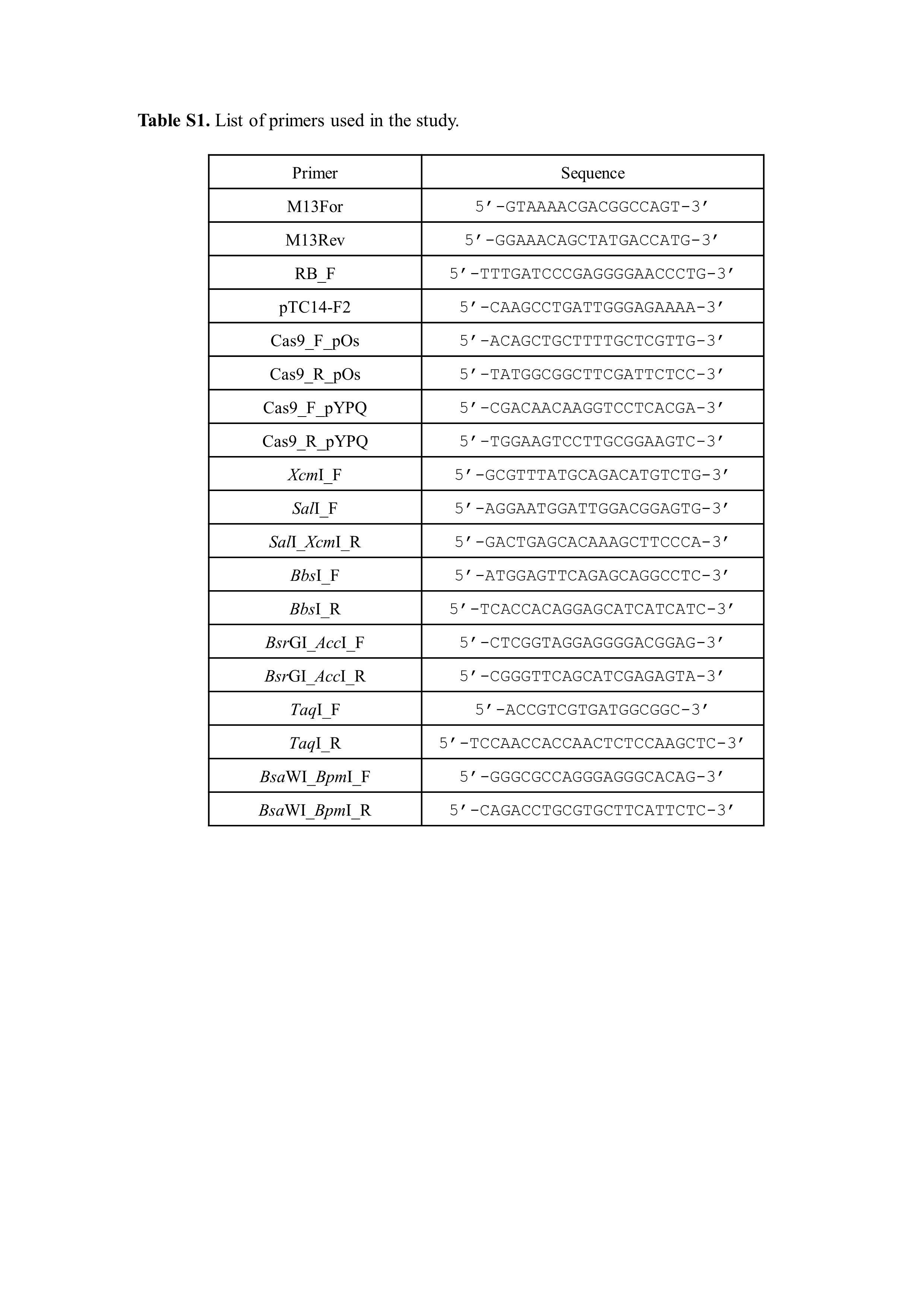

Supplement: Supplementary file 1 [file Image_1.tif]

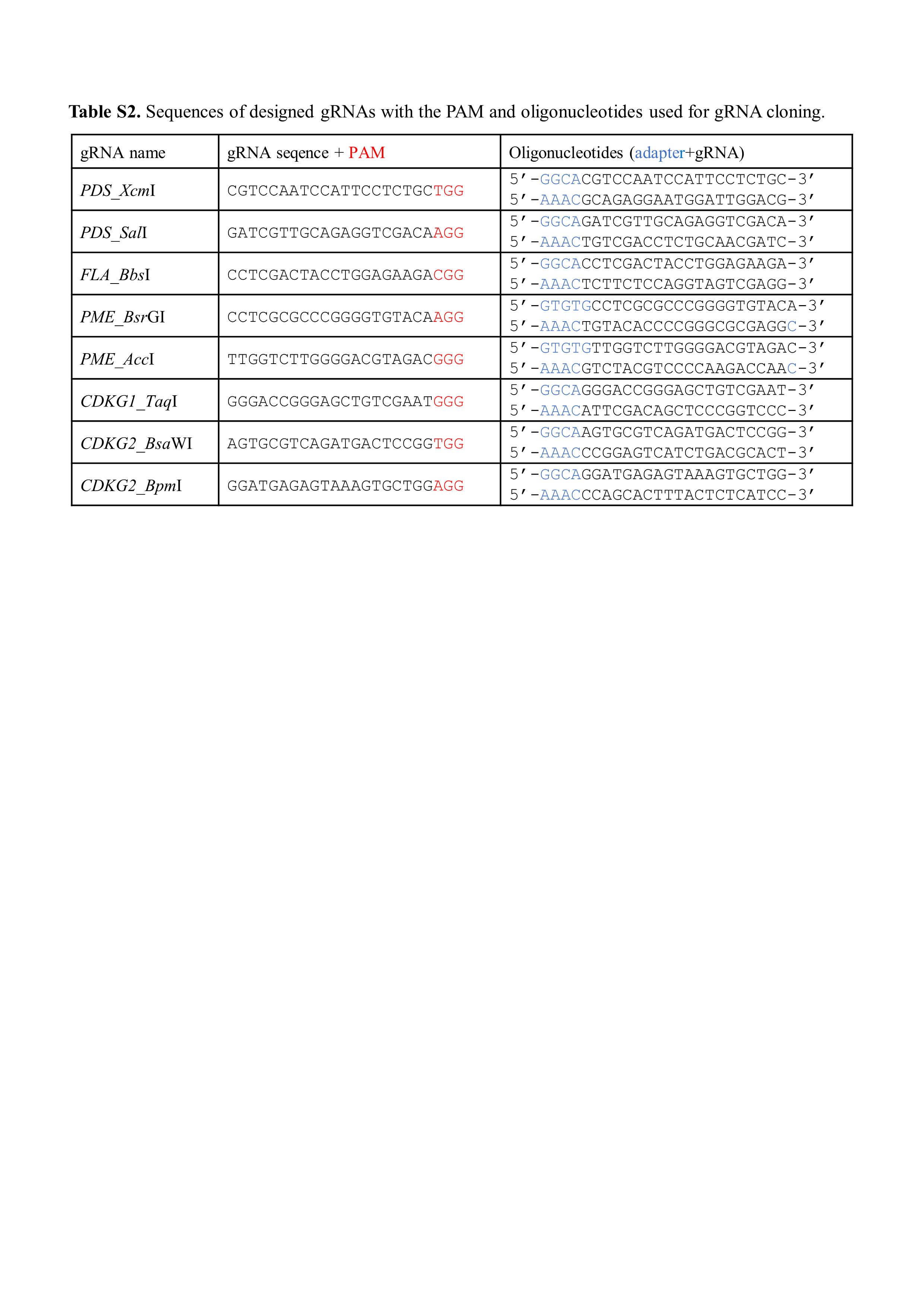

Supplement: Supplementary file 2 [file Image_2.tif]
